# Polyphenols as Antioxidants for Extending Food Shelf-Life and in the Prevention of Health Diseases: Encapsulation and Interfacial Phenomena

**DOI:** 10.3390/biomedicines9121909

**Published:** 2021-12-14

**Authors:** Marlene Costa, Zerrin Sezgin-Bayindir, Sonia Losada-Barreiro, Fátima Paiva-Martins, Luciano Saso, Carlos Bravo-Díaz

**Affiliations:** 1 REQUIMTE/LAQV, Department of Chemistry and Biochemistry, Faculty of Science, University of Porto, Rua do Campo Alegre 687, 4169-007 Porto, Portugal; marlene.andreia.costa@gmail.com (M.C.); sonia@uvigo.es (S.L.-B.); mpmartin@fc.up.pt (F.P.-M.); 2 Departamento de Química-Física, Facultad de Química, Universidad de Vigo, 36310 Vigo, Spain; 3 Department of Pharmaceutical Technology, Faculty of Pharmacy, Ankara University, Ankara 06560, Turkey; Zerrin.Sezgin@pharmacy.ankara.edu.tr; 4 Department of Physiology and Pharmacology “Vittorio Erspamer”, Sapienza University of Rome, 00185 Rome, Italy; luciano.saso@uniroma1.it

**Keywords:** polyphenols, free radicals, encapsulation, drug delivery systems, oxidative stress, biointerfaces

## Abstract

Toxicity caused by the exposure to human-made chemicals and environmental conditions has become a major health concern because they may significantly increase the formation of reactive oxygen species (ROS), negatively affecting the endogenous antioxidant defense. Living systems have evolved complex antioxidant mechanisms to protect cells from oxidative conditions. Although oxidative stress contributes to various pathologies, the intake of molecules such as polyphenols, obtained from natural sources, may limit their effects because of their antioxidant and antimicrobial properties against lipid peroxidation and against a broad range of foodborne pathogens. Ingestion of polyphenol-rich foods, such as fruits and vegetables, help to reduce the harmful effects of ROS, but the use of supramolecular and nanomaterials as delivery systems has emerged as an efficient method to improve their pharmacological and therapeutic effects. Suitable exogenous polyphenolic antioxidants should be readily absorbed and delivered to sites where pathological oxidative damage may take place, for instance, intracellular locations. Many potential antioxidants have a poor bioavailability, but they can be encapsulated to improve their ideal solubility and permeability profile. Development of effective antioxidant strategies requires the creation of new nanoscale drug delivery systems to significantly reduce oxidative stress. In this review we provide an overview of the oxidative stress process, highlight some properties of ROS, and discuss the role of natural polyphenols as bioactives in controlling the overproduction of ROS and bacterial and fungal growth, paying special attention to their encapsulation in suitable delivery systems and to their location in colloidal systems where interfaces play a crucial role.

## 1. Introduction

### 1.1. Oxidative Stress

Oxidative stress has been closely associated to the development of pathogenesis and a large variety of diseases [1,2,3,4,5]. Particularly, the overproduction of reactive oxygen and nitrogen species (ROS and RNS, respectively) has been frequently evoked as the main cause of various diseases, including age-related and metabolic disorders, cancer, diabetes, and cardiovascular diseases, among others [1,3,6,7]. At the chemical level, the term ROS and RNS refer to a group of short-living radical and non-radical species with different chemical reactivity, produced by the action of oxygen inside and outside of cells. At the enzymatic level, species such as those of superoxide dismutases (SOD), catalases, peroxiredoxins, sulfiredoxins, and glutathione peroxidases are also involved in the formation of ROS that can be harmful if not properly controlled [7,8]. Table 1 collects some of the most common ROS and RNS. For the sake of simplicity, only the reactions with reactive oxygen species (ROS) are reviewed here.

**Figure 1:**
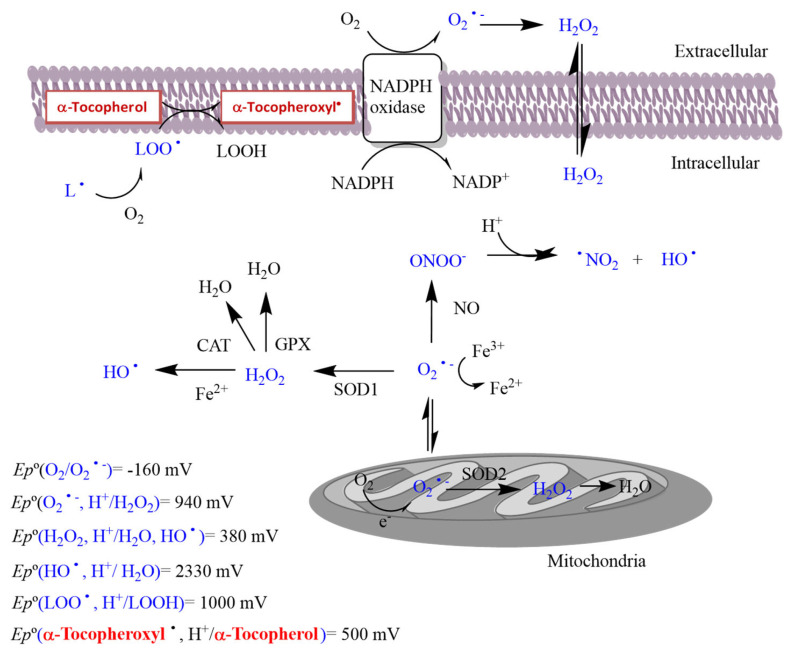
Reactive oxygen and nitrogen species (ROS and RNS) generation within a cell, and standard redox potentials (pH = 7) for the redox couples involved in the formation of some ROS, and in their inhibition, by radical scavengers [8]. CAT: catalase, SOD: superoxide dismutase, GPX: glutathione peroxidase, NADPH oxidase: nicotinamide adenine dinucleotide phosphate oxidase.

ROS can be produced as a metabolic by-product and, when in low concentrations, they have been proved to be necessary and beneficial, due to their signaling effects [1,3,9,10,11]. When in higher concentrations, ROS can lead to a significant modification of the cellular redox homeostasis, leading to a redox imbalance, which has detrimental consequences on human health. For example, an excess of ^•^OH radicals and ONOO- can produce lipid oxidation, damaging cell membranes and lipoproteins, as seen in Figure 1. However, the changes in the cellular redox equilibria cannot explain, by themselves, the overall consequences of the oxidative stress. Thus, a full rationalization of oxidative stress’ effects need to incorporate aspects including the chemical nature of the ROS, their location in tissues, the kinetics of their formation and degradation, the time of exposure, and the quantitative detection of increases or decreases in ROS levels [5,9].

ROS are formed through electron-transfer reactions whose redox potentials, *E_p_*^0^, depend on their environmental conditions. Molecular oxygen is a poor univalent electron acceptor (ergo a poor oxidant), with a redox potential of −0.16 V (referred to the standard state of [O_2_] = 1 M and pH 7) [1]. The addition of electrons leads to the formation of a number of oxygen-centered radicals and oxygen-centered nonradicals [2,3], and the one-electron redox potentials in water of some ROS are given in Figure 1 [4].

Table 2 and Table 3 show the broad range of reactivities of ROS, which are closely linked to their redox potential values, Figure 1, due to the low activation energies of the reactions; meanwhile, those of the non-radical ROS are much slower, and depend on the activation energy values. In cellular systems, ROS diffuse to different extents and estimations of the distance they travel prior to reacting contribute to understanding their effects. This distance can be estimated by considering the relationship between the mean square displacement of the various ROS species, their diffusion coefficients, and their lifetimes τ (Einstein equation) [8]. Estimated travel distances suggest that ^•^OH radicals have short half-lives in solution (t_1/2_ = 10^−9^s, Table 1) and react with nonspecific targets at a distance of a few nanometers, meanwhile H_2_O_2_ or O_2_^•−^ may spread a few tens of micrometers, exerting a longer range of effect. Further information on the chemical properties of ROS, together with thermodynamic and kinetic data, can be found elsewhere [8] and in references therein.

To control for the redox imbalance by reducing ROS levels, radical scavengers as antioxidants (AOs) are a feasible systematic treatment, as they are not expected to have any possible side effects [3,8,15,16]. Polyphenolic antioxidants are widely used in preventive medicine and a number of cohort and human epidemiological studies suggest that there is an inverse relationship between polyphenol intake and antioxidant levels, which is usually associated with the routinely intake of polyphenol-rich foods [1,4,15,17,18,19,20].

### 1.2. Physiological Functions of ROS

It was believed for a long time that an increase in the formation of ROS was closely associated to oxidative stress, representing an unavoidable biochemical problem associated to aerated life, that can be somehow prevented through a systematic antioxidant treatment [1,2,3,6,7]. This misconception is usually associated to the incorrect assumption that ROS has no physiological functions, but only react with relevant tissues and cell components with harmful effects [1]. ROS are now considered not necessarily as unsafe or dangerous, molecules but rather as essential signaling messengers. The distinction between the beneficial and detrimental effects of ROS is difficult and not always clear, but a good starting point can by considering the nature of the ROS that are formed, and how they are formed (physiological or non-physiological) in different cellular and subcellular compartments [4,9,10,11,21,22].

It is commonly accepted now that ROS are implicated in the regulation of aging because of the increases in the production of ROS, but there is no consensus that this ROS-induced damage is the underlying cause of aging [23,24]. The initial studies supporting the theory that the deleterious effects of ROS come from the increasing of hypoxia during the replication of human diploid fibroblasts with an increasing lifespan (theory of aging) [25]. However, recent theories suggest that an increase in ROS levels can activate cellular stress pathways to reduce tissue degeneration, and that such an increase may be beneficial for the maintenance of tissues [1,3,23].

As early as in 1970, researchers found that lipid peroxidation is increased during exercise in humans and rats [23,26]. A few years later, researchers began to link ROS and muscle function, finding that ROS are involved in normal muscle contraction [27,28]. Reid et al. [27] reported that, under basal conditions, low levels of ROS need to be present in skeletal muscles for their contraction, but antioxidant-mediated depletion of ROS prevents their contraction, concluding that ROS that are produced during exercise have a physiological role, signaling a modulated response of muscles to exercise [26,28,29]. In working muscles, the generation of ROS as part of a beneficial physiological response is mainly performed in the mitochondria, sarcolemma, and microcirculation [4]. The addition of antioxidants, such as vitamin C and E, appear to blunt exercise-induced mitochondrial biogenesis, as seen in Figure 1 [30,31]. Other reports suggest that the intake of vitamin C supplements decreases oxidative stress and might increase exercise performance only in those subjects with a low initial concentration of vitamin C [32]. It is believed that the adaptations of muscles depend on the strength and endurance required for exercise, and that these potential positive effects may not only be linked to the exercised muscles, reducing fatigability and improving recovery, but also alleviating common cold symptoms and exercise-induced bronchoconstriction, problems commonly associated with the upper respiratory system [33].

ROS also have physiological effects in excitation–contraction protein processes within cardiac muscles [34,35]. Several researchers found that ROS permit the regulation of Ca^2+^ signaling sensitivity through redox modifications, facilitating faster Ca^2+^ release during cardiac activity [36]. ROS have also a role in encoded cell death and cancer, because of their involvement in cell death processes. However, ROS overproduction may also be involved in the defense of the host organism, by attacking the DNA of cancer cells [23].

Changes in oxygen concentration (oxygen homeostasis) are controlled via both respiratory ventilation and red blood cells [23]. Increasing evidence suggests that changes in [O_2_] may be perceived independently by different ROS-producing proteins, including cytochrome b. Some literature reports indicate that ROS are involved in the control of red blood cell mass and ventilation so that during an ischemia event, ROS limit erythropoietin protein production, preventing both hypertension and the augmentation of blood viscosity. ROS are also involved in the amplification of immune responses, which are redox-regulated processes regulated through the activation of T lymphocytes or by a shift in the intracellular glutathione redox state. Recent research has focused on the later stages of the immune response in disease, suggesting that a moderate increase in the ROS levels of the immune system might enhance normal immune function [23].

### 1.3. Antioxidants and Their Modes of Action

A conceptually important strategy to slow down the harmful effects of ROS is stimulated by the endogenous security processes developed by biological systems to maintain the redox homeostasis, so that they can protect themselves against induced cell damage. This approach involves the use of chemicals that scavenge ROS, blocking their production, chelating pro-oxidant transition metals, inhibiting enzymes associated with the overproduction of ROS, and recovering the antioxidant defenses by addition of exogenous antioxidants [8,37]. Typical endogenous antioxidants that are involved in some of their reactions are displayed in Table 4. Antioxidants (either endogenous or exogenous) are effective as free radical scavengers because they are capable of donating H-atoms to counteract the adverse effects of ROS [38] and they work at distinctive levels, keeping the formation of reactive species to a minimum, and scavenging ROS by using non-catalytic and catalytic molecules, e.g., alpha-tocopherol and ascorbic; this can also be achieved by repairing damaged molecules, for example, regenerating antioxidants or lipid radicals to their original species [38,39,40].

Polyphenolic antioxidants can be divided in two broad groups according to their mode of action against ROS. Primary, chain-breaking antioxidants reduce or delay the production of ROS, usually by trapping the generated free radicals. The secondary or preventive antioxidants prevent the attack of ROS on a substrate. Primary (chain-breaking) antioxidants inactivate free radicals through three main mechanisms that can operate simultaneously, depending on the particular environmental conditions: (1) transferring H-atoms to peroxyl radicals (the hydrogen atom transfer, HAT, mechanism); (2) the sequential electron transfer-proton transfer (SETPT mechanism); and (3) the sequential proton loss-electron transfer (SPLET) mechanism [38,39,41]. Some kinetic and mechanistic details can be found elsewhere [8].

**Scheme 1:**
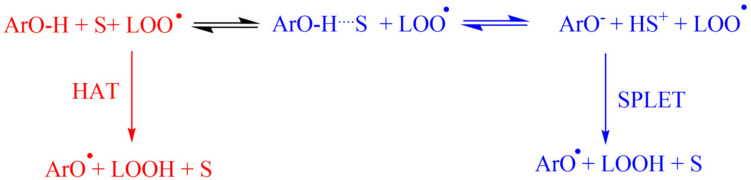
Hydrogen atom transfer (HAT) and single-proton-loss electron-transfer (SPLET) reaction mechanisms between antioxidants and peroxyl radicals. ArO-H: antioxidant, LOO^•^: peroxyl radical, S: solvent.

**Scheme 2:**
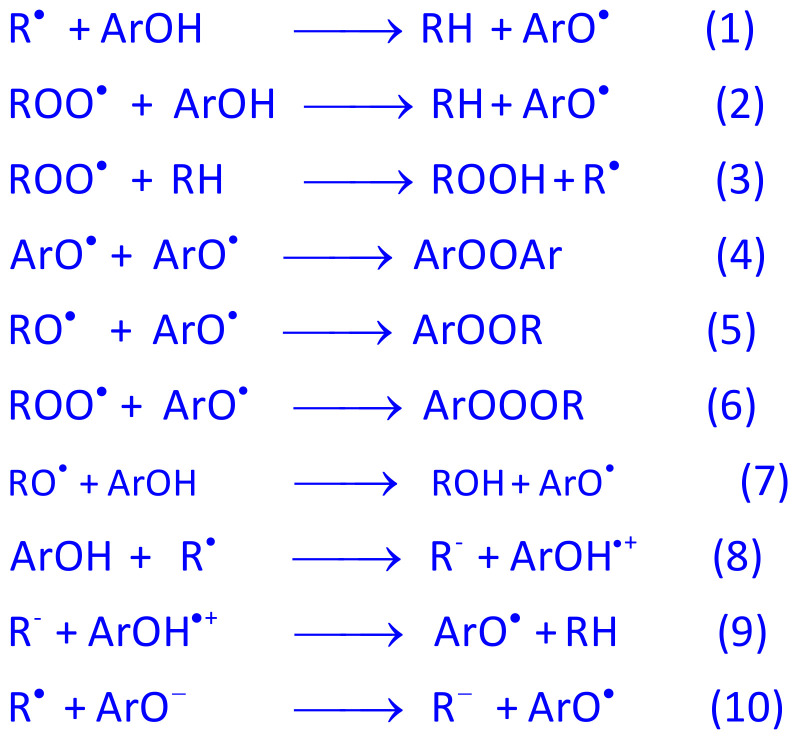
Key reactions of lipid radicals (R^•^, RO^•^ or ROO^•^) with antioxidants (ArOH) and antioxidant radicals (ArO^•^).

The HAT mechanism involves the hemolytic cleavage of the O-H bond, converting them into harmless hydroperoxides and the oxidized antioxidant radical ArO^•^, which is less reactive than ROS. The so formed ArO^•^ radicals are not capable of triggering the formation of fresh radicals, interfering in the free radical propagation pathways, as seen in Scheme 1, but can accept electrons from other radicals to form stable, non-radical products (SETPT, SPLET mechanisms, Scheme 1 and Scheme 2) [41,42,43].

The so-called preventive, or secondary, antioxidants retard oxidation through forming thermodynamically stable complexes with pro-oxidative metal ions (ethylenediaminetetraacetic acid (EDTA), citric and phosphoric acids), enzymes, and other promoters [44,45]. Synergistic or antagonistic effects may be observed when two or more antioxidants (AOs) are present at a time, so that their scavenging reactivity is much higher or lower than that of when used alone [44,45]. For example, the couple α-tocopherol/ascorbic acid is synergistic in the inhibition of the lipid oxidation reaction, because tocopherols (primary antioxidants) are replenished from their radicals by ascorbic acid, which is capable of donating hydrogen atoms, inhibiting its depletion [46,47]. Other species that may recover antioxidants include low-molecular-weight thiols and thioethers (methionine, glutathione, acetylcysteine, etc.).

### 1.4. Ongoing Development of Antioxidants

Addition of antioxidants may also have some effects of health. A large number of reports have been published on the protective and therapeutic benefits of antioxidants in cellular and animal models of cardiovascular, neurodegenerative, inflammatory diseases, and cancer [48]. Consequently, antioxidant supplements have been considered, often with little or no clinical control or evidence, either as nutraceuticals or antioxidant vitamins [4].

Many in vivo animal studies have failed to show their benefit in the clinic, in spite of the beneficial effects shown by antioxidants [4]. However, a large number of epidemiological studies have unequivocally associated the consumption of antioxidants (polyphenols) with a decrease in the risk of development of diseases, including atherosclerosis, cancer, and neurodegenerative processes [49]. Thus, current antioxidant therapies have focused on polyphenolic compounds, which donate H-atoms to radical species to minimize radical concentrations.

One potential important problem in interpreting the cytoprotective effects of antioxidants relies on the chosen assays to monitor their action and on the systems where the effects of ROS are monitored. Standard cell-free, cell culture, and isolated organs model systems may bias results if experimental conditions do not reproduce those present in real systems. For example, the exposure of cells to high concentrations of antioxidants is certainly non-physiological, even though they may be reached in some subcellular compartments or in intercellular spaces [4,46]. Numerous in vitro assays have evaluated the antioxidant activity of biological samples, but establishing comparisons between them is difficult because of the different chemical assays involved (see Section 3.2).

Currently, the distribution of antioxidants is recognized as a crucial issue regarding their efficiency at inhibiting the oxidation of target biomolecules in multiphasic systems. Current research links the quantitative distribution of antioxidants in complex multiphasic systems with the selection or design of antioxidants that preferentially distribute at the bio-interfaces [18,47,50].

## 2. Antibacterial Activity of Polyphenolic Antioxidants: Interactions with Cell Membranes

**Scheme 3:**
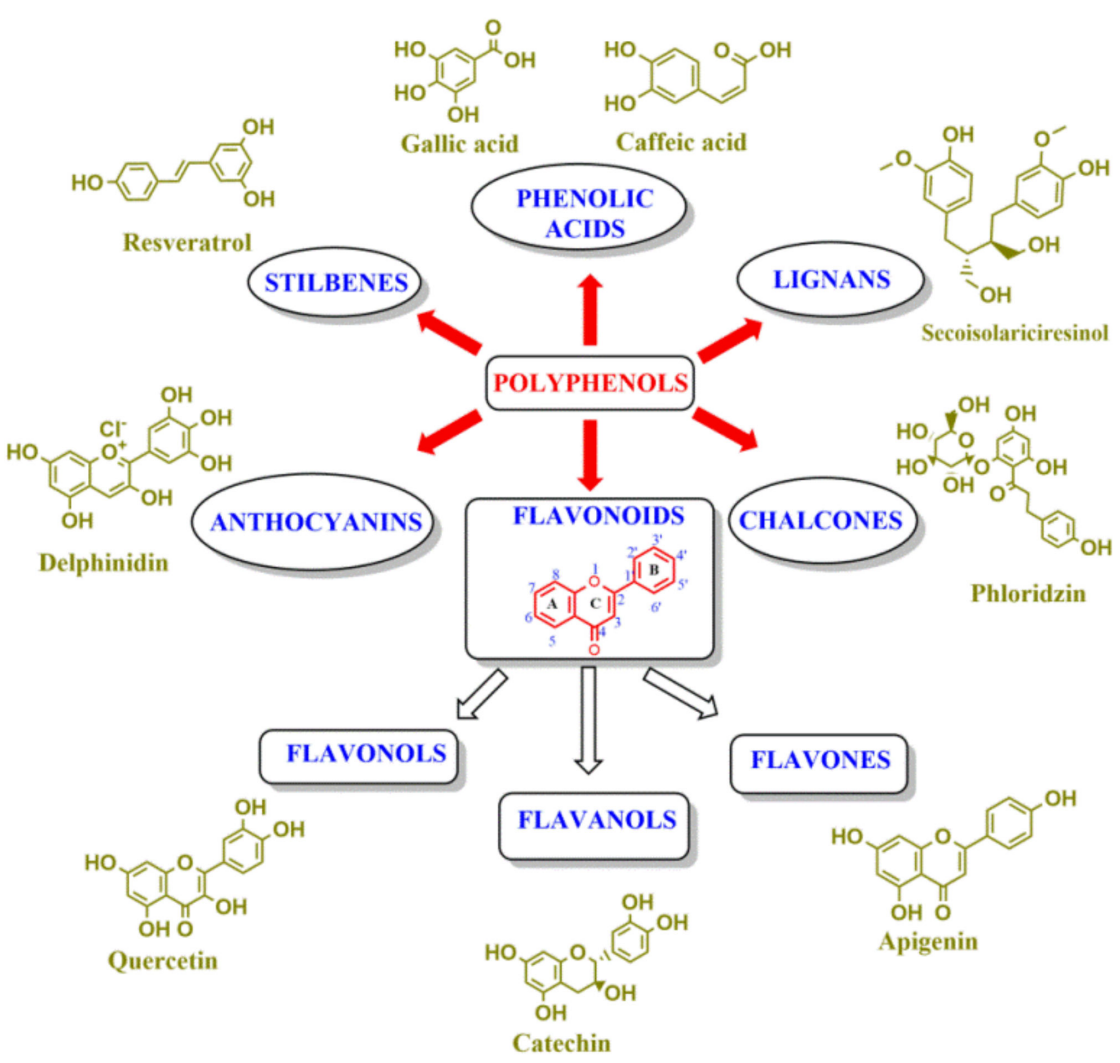
Chemical structures of some common antioxidants with antibacterial and anticarcinogenic activity. Adapted from ref. [8].

In addition to their beneficial effects on human health, phenolic compounds also play a significant role in defense mechanisms against viruses, bacteria, fungi and herbivores, making them behave as natural preservatives in agriculture and in the meat industry. Phenolic antioxidants are chemical compounds, bearing at least an aromatic ring with one or more attached hydroxyl groups, and several thousands of naturally occurring species, from simple phenolic acids to highly polymerized compounds, have been described [51,52]. The major groups of phenols that show antimicrobial activity include phenolic acids, coumarins, quinones, flavonoids, and tannins, as seen in Scheme 3, and variations in their architecture and spatial configuration results in differential antimicrobial action.

**Scheme 4:**
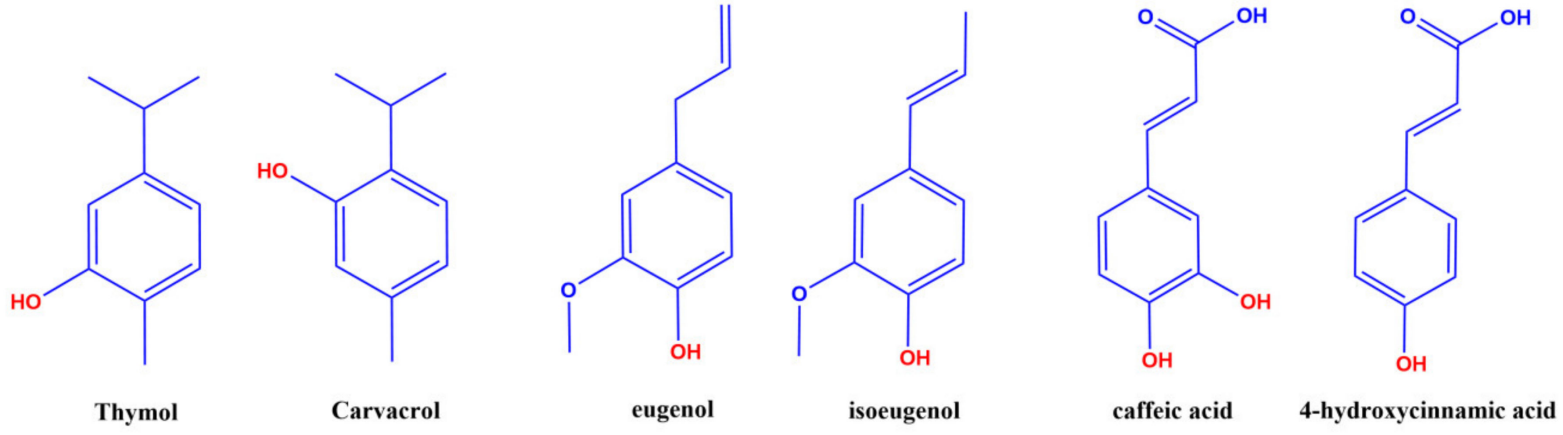
Changes in the number and position of substituents in the aromatic ring (thymol-carvacrol, eugenol-isoeugenol, caffeic acid—4-hydroxycinnamic acid) lead to changes in antibacterial effectiveness.

The mechanism underlying the antibacterial activity of polyphenols is still a matter of debate. Some researchers have argued that the antibacterial ability of polyphenols is related to their ability to chelate metals, especially iron, which is vital for the survival of almost all bacteria; however, others think that the inhibitory action comes from the interaction of the hydroxyl (-OH) group in phenolics with the cellular membranes of bacteria, disrupting their membrane structures and causing the leakage of important cellular components, as seen in Scheme 4. The strength of the interaction not only depends on the number and position of the -OH groups in the aromatic ring, but also depends on the nature and complexity of the bacterial cell wall [53]. For instance, Gram-positive bacteria are less resistant to plant extracts, oils, and their constituents than Gram-negative bacteria, because the cell wall of Gram-negative bacteria is more complex [54]. Hydrophobic molecules penetrate easily through the cell walls of Gram-positive bacteria, acting on both the cell wall and in the cytoplasm [55], meanwhile only small, hydrophilic solutes can penetrate the cell walls of Gram-negative bacteria, and this is one of the reasons why Gram-negative bacteria are more resistant to polyphenolic action [56].

It is believed that polyphenols interact with the outer bacterial membrane through non-specific forces, including hydrogen bonding and hydrophobic and lipophilic effects, [57] leading to physical interactions with the phospholipids of the lipid bilayer and with the membrane proteins [58]. For example, in bacteria (both Gram-positive and Gram-negative) the physical interactions of polyphenols with membrane proteins disrupts the lipid bilayer and perturbing membrane fluidity, causing increases in membrane permeability and modifying ion transport processes [57].

The number and position of -OH groups in the aromatic ring and the length of the saturated/unsaturated side chain are also factors influencing antimicrobial activity [59]. Gallic and ferulic acids impact irreversibly the membrane properties of *E. coli*, *S. aureus*, *P. aeruginosa*, and *L. monocytogenes* [60]. It was postulated that gallic and ferulic acids produce a decrease in the (negative) surface charge of bacterial cytoplasmic membranes, modifying their hydrophobicity and leading to the formation of pores that allow the leakage of intracellular constituents [61]. Attachment of alkyl or alkenyl groups to the aromatic ring also affects the antimicrobial activity of phenolic compounds. For instance, benzaldehydes with two or more adjacent -OH groups have been reported to be more active than aldehydes, as benzaldehydes are thought to act primarily on the external surface of the cells, combining with sulphydryl groups of proteins [62,63].

## 3. Bioactivity of Antioxidants

### 3.1. Bioavailavility of Phenolic Antioxidants

The bioavailability of phenolic antioxidants in humans is still not well understood, because before becoming bioavailable, antioxidants must be released from complex foods matrices and suffer several modifications in the gastrointestinal (GI) lumen. Therefore, any potential health effect obtained by the consumption of antioxidants is dependent on their effective bioaccessibility and bioavailability.

The bioavailabilities of phenolic antioxidants are quite different from each other, even within each class of phenols, as seen in Scheme 3, and frequently the most abundant antioxidants in foods are not necessarily those with the best bioavailability or bioactivity in humans. The physicochemical characteristics of antioxidants, such as their structure, molecular weight, and glycosylation will impact their solubility and are critical factors in their bioavailability. Small molecules such as hydroxytyrosol, flavonoids, or phenolic acids are usually easily absorbed in a dose-dependent manner [64] and are detected in plasma as parental compounds or as their metabolites, within 5 h after consumption [60,65,66]. In contrast, polymeric polyphenols, such as proanthocyanidins, are poorly absorbed [60,67]. Moreover, phenolic rings with more hydroxyl and less methoxy groups show, in general, a lower bioavailability [68].

**Figure 2:**
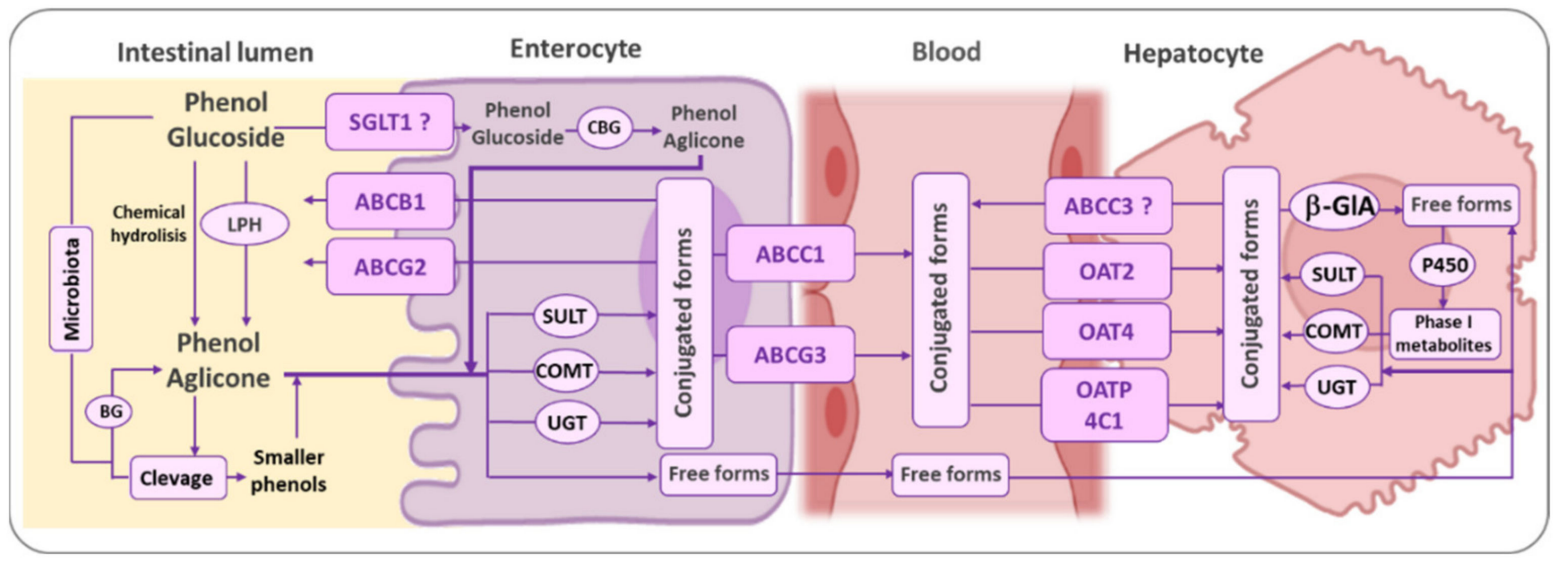
Illustration of the complex in vivo polyphenol metabolism. In the intestinal lumen, phenolic glucosides are hydrolyzed by lactase phloridzin hydrolase (LPH) in the brush border of the enterocyte. The phenolic aglycone is able now to enter the enterocyte, and is conjugated by uridine diphosphate glucuronosyl transferase (UGT) or sulfotransferase (SULT) enzymes. The conjugated metabolites can then be sent back to the intestinal lumen or sent into the bloodstream through several ATP-binding cassette transporters (ABC). Conjugated metabolites can be taken by hepatocytes through a series of uptake transporters (OAT transporters) and then send back to the circulatory system or they can be deconjugated in the cell by enzymes such as β-glucuronidase (β-GlA) and suffer further metabolism before being sent to the bloodstream. BG, β-glucosidase; CBG, cytosolic BG; SCLT1, sodium-dependent glucose transporter; OAT, organic anion transporter; OATP, organic anion transporting peptide.

After ingestion, antioxidants must pass from the gut lumen into the circulatory system in order to be absorbed. Due to the special acid environment of the stomach, the more liposoluble phenols can cross the gastric mucosa, mainly by passive diffusion [69,70], but due to relatively low time of residence, the absorption in the stomach is considered relatively low and untimely; their absorption will happen to a greater extent in the small intestine. In nature, phenols are usually in the form of glycosides, which substantially decreases their liposolubility, and prevents their transport through membranes. Therefore, usually the attached sugar must be hydrolyzed before absorption [70]. This hydrolysis may take place at the brush border of the enterocyte (Figure 2) and is usually catalyzed by lactase phlorizin hydrolase (LPH), with the release of the much more lipophilic aglycone [71]. The aglycone is then able to enter the enterocyte by passive diffusion. Alternatively, glycosides can be transported into the enterocyte by active transport through a sodium-dependent glucose transporter (SGLT1) [72] And then be hydrolyzed by cytosolic β-glucosidases (CBG), as seen in Figure 2, inside the cell.

**Scheme 5:**
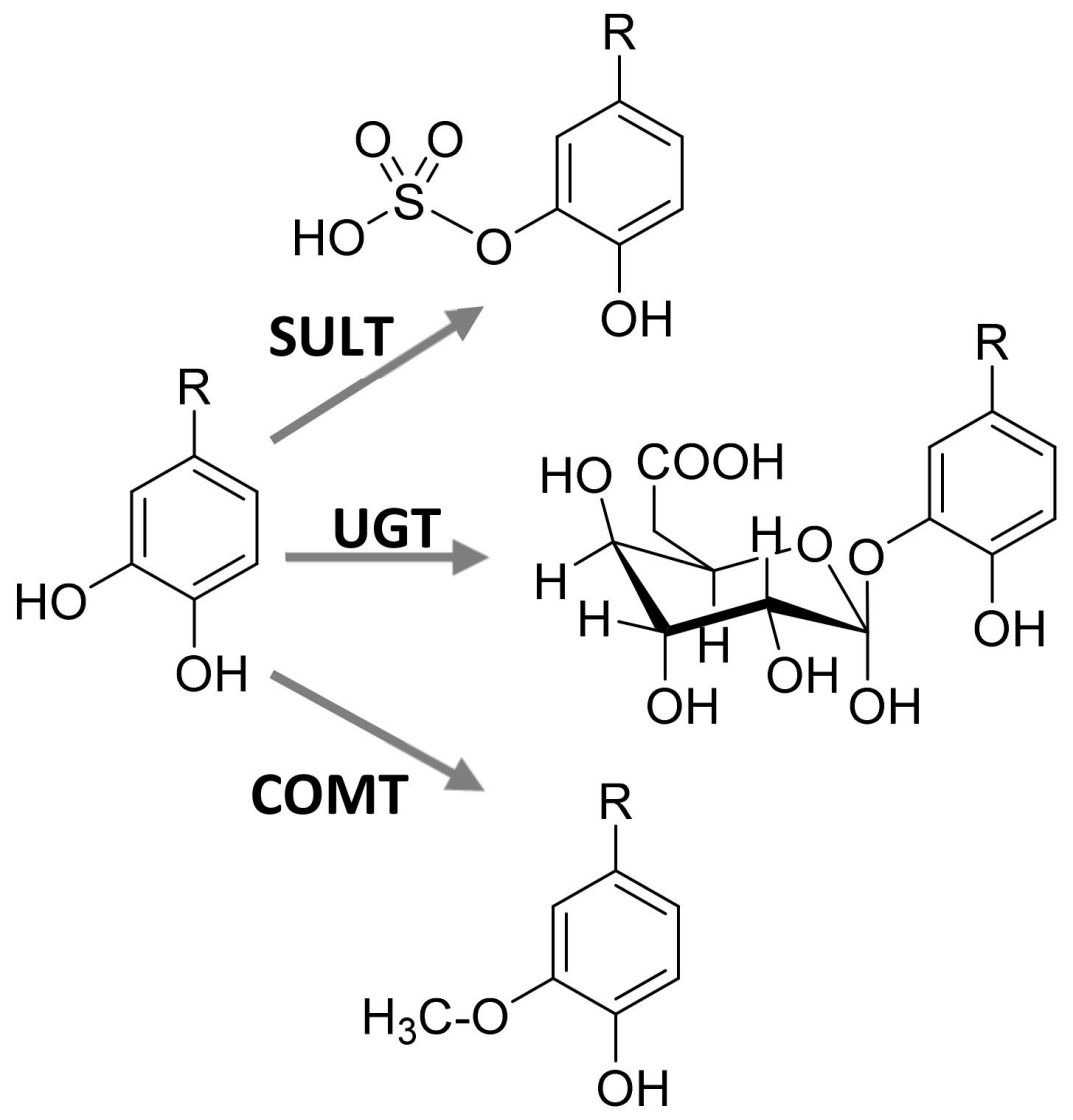
Main conjugation reactions of phenolic hydroxyl groups: glucuronidation, sulfonation, and/or methylation through the action of uridine-5-diphosphate glucuronosyltransferases (UDG), sulfotransferases (SULT), and catechol-O-methyltransferases (COMT).

Dozens of new compounds can arise from the metabolism of each phenolic compound, which makes the fate of each compound in the body difficult to follow [70]. These metabolic processes convert the phenolic antioxidants, in most cases, to completely distinct molecules, whereas the parental phenol cannot be detected [70]. Once inside the enterocyte, xenobiotics may first undergo a functionalization reaction (phase I metabolism) of oxidation, reduction, or hydrolysis, which introduces or exposes a functional group, such as a hydroxyl group, suitable for conjugation (phase II metabolism) [70,71]. In the case of phenolic antioxidants, the phenolic ring gives to these compounds some metabolic pathways that are shared by all classes of phenolic compounds [65,70]. In the enterocyte, usually the aromatic structures are kept untouched, but the free hydroxyl groups at the aromatic ring of phenols are efficiently conjugated with the production of glucuronides, sulfates, and/or methylated metabolites through the action of uridine-5-diphosphate glucuronosyltransferases (UDG), sulfotransferases (SULT), and catechol-*O*-methyltransferases (COMT) (Scheme 5) [69,73]. The catecholic antioxidants’ conjugation usually occurs preferentially at the *meta* position [70,74]. Nevertheless, conjugation with glucuronic acid or a sulfate group may also occur at the *para* position, usually after conjugation at the *meta* position with a methyl group. In contrast, derivatives of gallic acid may conjugate preferentially at the *para* position [73].

Nevertheless, further phase I metabolic reactions may still take place inside the enterocyte by the action of several enzymes, such as dehydrogenases, in the non-phenolic moiety of antioxidants.

Once conjugated by SULTs and UGT, the ability of compounds to cross membranes deeply decreases, due to the attached anionic hydrophilic moiety and specific transporters are now required [75]. Conjugated metabolites can be sent back to the intestinal lumen through specific efflux ATP-binding cassette transporters (ABC transporters) or sent to the bloodstream by other ABC transporters and reach the liver via the hepatic portal vein. Here, several organic anion transporters (OAT) families, namely the organic anion transporting peptide (OATP) family and the multidrug resistance-associated protein (MRP) family (ABC transporters), will allow the conjugated compounds to enter the hepatocyte. However, these transporters are quite specific for the class of phenols, to the type of conjugated group, and conjugated position in the molecule [76].

Once in the hepatocyte, some conjugates may be sent back to the bloodstream and distributed to other organs tissues or up taken into the kidney proximal tubular cells by transporters and excreted into the urine. In contrast, other conjugates can be deconjugated in the cell by enzymes such as β-glucuronidase (β-GlA) or sulphatases. Intracellular deconjugation is enhanced under inflammatory conditions, and seems to have an important role in the bioactivity of many conjugated metabolites. For example, there is growing evidence that intracellular desulfation plays an important role in the availability of active steroid hormones near the target sites [77]. In fact, the role of phase II conjugates as temporary deposits in tissues has recently attrated some attention [78,79].

In the liver there are two main metabolic reactions that are associated with the metabolism of xenobiotic compounds: conjugation, catalyzed by the enzymes UGA, SULT, COMT, as seen in Scheme 5, and also by glutathione-S-transferases and oxidation, reduction and hydrolysis reactions, mainly catalyzed by the cytochrome P450 enzyme system, a superfamily of membrane-bound isoenzymes. Nevertheless, a number of non-cytochrome P450-dependent reactions also occur in the liver. Moreover, although the majority of metabolic reactions occur in the liver, cytochrome P450 isozymes are presented in other tissues, including the gastrointestinal tract itself, the lungs, kidney, and skin. Therefore, each phenolic antioxidant may produce in the body a huge number of compounds, distributed in different concentrations over the different tissues and organs, depending on their capacity of being transported, and are then metabolized and eliminated by cells, with each of these metabolites being bioactive, weakly bioactive, reactive with endogenous macromolecules, or non-active at all.

It is estimated that only 10–15% of polyphenols are absorbed in the small intestine [76]. Therefore, most reach the large intestine and are subjected to the action of the intestinal microbiota, producing a wide number of metabolite compounds. Therefore, most of the observed bioactivity of antioxidants results from the metabolism performed by the gut microbiota, rather than the original dietary antioxidants [70]. A number of microbiota enzymes are able to hydrolyze glycosides, to catalyze ring fissions leading to the production of smaller phenolic compounds, and to oxidize or reduce the non-phenolic moieties of antioxidants [80,81]. These more lipophilic metabolites can then be absorbed, to be subjected to phase II metabolism and this may be responsible for a large extent of the bioactivity of antioxidants (Figure 2). Ring fusions are of particular importance in the case of flavonoids, as this class of phenolic antioxidants will produce small phenolic acids, sometimes with a higher bioactivity than the parental flavonoid. The microbiota is able to catabolize flavonoids into C6−C3 phenolic catabolites that, in turn, can be converted by two α-oxidations or via β-oxidation into C6–C1 phenolic compounds. C6−C2 phenolic catabolites usually arise independently, possibly by α-oxidation [82,83,84,85,86]. Many of these transformations can be catalyzed by both the microbiota’s and/or mammalian enzymes. Increasing evidence suggests that microbiota phenolic catabolism is responsible for much of the flavonoids bioactivity [87,88].

The importance of the microbiota to the bioactivity of antioxidants has gained huge interest in the last decade and it has shown to be one of the most important causes of interindividual variations that are observed in antioxidant bioavailability. Substantial interindividual variation has been observed in the excretion recovery of flavonoids and olive oil secoiridoids, ranging from 2 to almost 60% of the ingested phenolic compounds [89]. In fact, in many studies gender, body mass index, or even age and drug intake were not the primary cause of interindividual variations, but they may be related with the different intestinal microbiota of individuals [89,90]. Moreover, the presence of antioxidants seems to modulate the existing microbiota, suggesting their application in foods as probiotics [91].

### 3.2. Bioactivity of Phenolic Antioxidant Metabolites

As mentioned in Section 1.1, an imbalance between the generation of reactive oxygen and nitrogen species, namely hydroxyl radicals, superoxide anion radicals, hydrogen peroxide and nitric oxide, and antioxidant protection results in a disrupted condition called oxidative stress, which causes disturbances such as lipid oxidation, DNA mutation, and protein cross linking, which ultimately leads to cell malfunction, tissue damage, and necrosis. At a later stage, this will trigger oxidative stress associated with degenerative complications such as cancer, diabetes, as well as cardiovascular (CVD), liver and neurodegenerative (NDD) diseases. Hypertension, dyslipidemia, hyperglycemia, and obesity occur very often in people suffering from CVD, NDD, and diabetes, and these chronic conditions are usually intimately related with inflammatory conditions. Hence, natural antioxidant compounds with a free-radical scavenging capacity should be considered as powerful candidates for the prevention and treatment of these diseases.

In general, phenolic antioxidants and their metabolites are present in plasma and tissues at concentrations more than 50-fold lower than the pool of endogenous antioxidants and, therefore, their contribution to the overall body oxidative status is likely to be quite low when compared to other endogenous oxidizable substrates, including vitamin E, vitamin C, urate, tiols, bilirubin, proteins, enzymatic systems, and unsaturated fatty acids [76]. Moreover, phenol-conjugated metabolites have frequently shown a much lower radical scavenging capacity than the parental compounds and, unless some intracellular de-conjugation happens, they are quite inactive as antioxidants [92,93]. Nevertheless, it is known that a relatively low plasma concentration of some drugs that are given at regular low doses (for example aspirin) can confer health benefits to patients [94]. Therefore, it is possible that the regular low lifetime intake of dietary phenolic antioxidants results in an overall antioxidant protective effect. This phenomenon has been observed in clinical trials, showing that the short-term consumption of olive oil (50 mL/day) rich in phenols could change several oxidative stress markers [95,96], although the concentration of these phenols was lower than those required to show in vitro biological activity. In general, antioxidant actions on human physiology are moderate and, as food components, they should not be regarded as drugs. Usually, the possibility of accumulation in particular tissues and their link to cellular proteins and lipoproteins is not known for most phenolic antioxidants, and probably underestimated. In fact, food and its components are ingested throughout a lifetime, during which even modest antioxidant effects may become noteworthy. Moreover, due to the strong possibility of additive or synergetic effects between phenols, it is difficult to predict the real contribution of these compounds to human health. Actually, it is important that antioxidants are consumed at the normal concentration found in foods and as part of the natural protective food matrix. Phenolic compounds may act as pro-oxidants when consumed in high doses as supplements or in the presence of transition metal ions inside the human intestinal tract, where they may induce oxidative damage [97,98].

**Figure 3:**
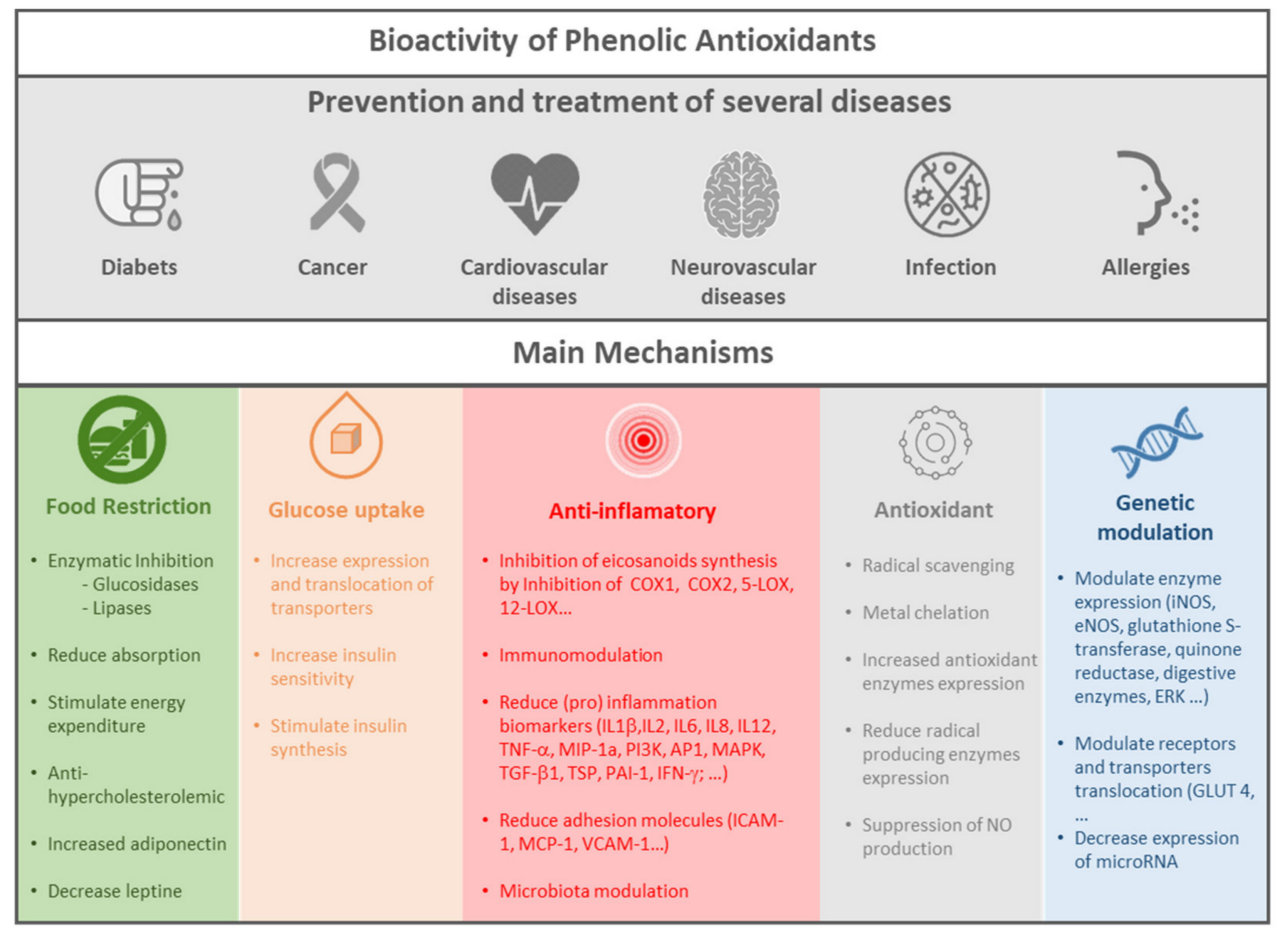
The bioactivity of phenolic antioxidants and some of their main mechanisms of action. Cyclooxygenase (COX), lipoxygenase (LOX), intracellular adhesion molecule-1 (ICAM-1), monocyte chemoattractant protein-1 (MCP-1), vascular cellular adhesion molecule-1 (VCAM-1), inducible nitric oxide synthase (iNOS), endothelial nitric oxide synthase (eNOS); tumor necrosis factor-α (TNF-α); extracellular signal regulated protein kinase (ERK); interleukin (IL), phosphoinositide 3-kinase (PI3K); transforming growth factor-β (TGF-β1).

There are several methods for evaluating the in vitro antioxidant activity of phenolic compounds, including the 2,2-diphenyl-1-picrylhydrazyl (DPPH) essay, the ferric reducing antioxidant power (FRAP), the oxygen radical absorbance capacity (ORAC), or even cell-based antioxidant assays [18]. However, the radical scavenging activity of antioxidants may vary against different oxidants. For instance, some phenols may exhibit a high radical scavenging activity against hydroxyl radicals, but a low capacity against superoxide anions [99]. Furthermore, monophenols do not show radical scavenging activity with several radicals used to evaluate in vitro radical scavenging activity, such as the DPPH^•^ [92]. However, in vivo, highly reactive radicals, such as the hydroxyl radical, are formed and can be trapped by monophenols in a similar fashion to the amino acid tyrosine that is present in all membrane proteins. Although the assessment of antioxidant activity using these in vitro tests is more or less quick, it has been clear that the bioactivity of phenolic antioxidants in vivo cannot be related to their in vitro antioxidant capacity without considering their bioaccessibility and bioavailability. Thus, in 2012, the USDA’s Nutrient Data Laboratory removed the ORAC Database for Selected Foods. Nevertheless, although the mechanism of action of phenolic antioxidants is still unrevealed, the underlying idea that phenolic antioxidants protect humans against oxidative stress remains valid [76], as diets which are deficient in natural phenolic antioxidants confer a strong risk factor for developing many chronic diseases [100]. Moreover, several interventional studies have linked the consumption of phenolic antioxidants with a lower prevalence of the most common chronic diseases such as diabetes type II, and cancer, as well as cardiovascular and neurovascular diseases, Figure 3.

Polyphenols have multiple therapeutic targets and bioactivities, as seen in Figure 3. Inside the gut they can reduce caloric intake by inhibiting digestive enzymes [101,102,103], such as β-amylase, β-glucosidase, and lipase, which prevents the increase in blood triacylglycerols and glucose concentration, and modulates the intestinal microbiota by favoring beneficial bacteria.

After being absorbed into the bloodstream, some metabolites are still able to scavenge reactive oxygen [92,93]. However, phenolic antioxidants led to the reduction of oxidative stress not only by scavenging ROS, but mostly by genetic modulation, as seen in Figure 3. In fact, phenolic antioxidants are capable of reducing the expression of ROS-generating enzymes/pathways, and increasing the expression and activity of antioxidant enzymes [104]. In addition, phenols also modulate the expression and activity of enzymes involved in lipid metabolism, such as the pancreatic lipase and carnitine palmitoyltransferase (CPT), affecting the concentrations of cholesterol and triacylglycerides in the blood [105,106]. Phenolic antioxidants are also able to increase the expression, translocation, and activity of glucose transporters and modulate the pathways of carbohydrate metabolism, lowering the blood concentration of glucose [107]. Moreover, they are also able to reduce body weight by decreasing the concentration of leptin and the expression of adipogenesis-activating genes, and by increasing the amount of adiponectin and the expression of thermogenesis genes [108], as seen in Figure 3. Other genetic modulatory activity has been attributed to phenolic compounds, such as the up-regulation of the CB₁ tumor suppressor gene in human colon cancer cells [109].

Many other mechanisms of action have been addressed to antioxidant phenolic compounds, one of them being their capacity to modulate the host immune cells’ response through binding to cell receptors and the modification of intracellular signaling pathways, as well as the induced expression of genes, as seen in Figure 3. Some have shown to be able to elevate the concentration of immune cells, namely, dendritic cells, macrophages, neutrophils, T helper 1 cells, natural killer (NK) cells, and B cells [110], which are beneficial for infectious disease prevention. NK cells have a key role in the body’s defense against cancer cells. On the other hand, some phenolic antioxidants have also shown the capacity to inhibit the release of histamine from mast cells [111] and to increase regulatory T cells, that have a key role in immunotolerance and prevent the development of immune diseases, such as allergies, rheumatoid arthritis, type I diabetes, and Crohn’s disease [112].

The most important cells in inflammatory responses, critical for immune diseases, are probably macrophages. These cells initiate inflammatory responses through the production of various pro-inflammatory mediators such as cytokines, prostaglandin E2, IL-6, and tumor necrosis factor α (TNF-α), but they also produce those with chemotactic activity, chemokines (Figure 3). Many phenolic antioxidants have shown, both in vitro and in vivo, to suppress the expression, production, and secretion of inducible nitric oxide synthase (iNOS), cyclooxygenase-2 (COX-2), TNF-α, and IL-1-β [113]. Moreover, the consumption of selected phenols has shown in patients with a high risk of cardiovascular disease (CVD) to reduce E-selectin, monocyte chemoattractant protein-1 (MCP-1), intracellular adhesion molecule-1 (IDM-1), IL-16, CD40 antigen and ligand, and vascular cellular adhesion molecule-1 (VCAM-1) [114].

One of the main anti-inflammatory, anti-thrombotic, and anti-hypertensive mechanisms of action of many phenols is the inhibition of eicosanoid biosynthesis, including PGE2, leukotriene B4 (LTB4), and thromboxane B2 (TXB2) [115]. COX-1 and 12-LOX pathways produce arachidonic acid radical derivatives and, therefore, antioxidants may inactivate the production of these mediators through a radical scavenging mechanism based on their reaction with the tyrosyl radical in COX-1, and the reduction of iron (III) to iron (II) in 12-LOX. Nevertheless, in spite of their lower radical scavenging capacity, the anti-inflammatory activity of phenols through the modulation of COX-1 and 12-LOX pathways does not seem to depend on the number of OH groups, as some monophenols, such as olocanthal, a tyrosol derivative from olive oil, demonstrate a strong anti-inflammatory activity. The apparently contradictory observations in the variation of the anti-inflammatory capacity of compounds with a different number of phenolic hydroxyl groups may be related to differences in the interaction of compounds with the different active sites of enzymes. Nevertheless, and whatever the mechanism, it seems clear that many phenolic antioxidants are able to effectively modulate hemostasis, as well as endothelial and platelet function, and inflammatory biomarkers [110].

The intensity of the bioactivity of phenols depends heavily on their structure. For example, in order to show immunosuppressive activity, flavonoids need to hold a methoxyl group at the C’4 position and a hydroxyl group at the C-5 position, as seen in Scheme 3. This activity is usually increased by the presence of hydroxyl groups at both 3′-and 4′-positions in the B-ring and is higher than the one observed for those with 3′-,4′- and 5′-OH groups but, as already mentioned, having more than a phenolic hydroxyl group is not always needed. Glycosides usually have a weaker activity than their aglycones, but their immunosuppressive activity also depends on the size, position, type, and number of glycoside sugar moieties [110]. Nevertheless, the anti-inflammatory activity of phenols through the modulation of COX-1 and 12-LOX pathways, as mentioned above, does not seem to depend on the number of phenolic OH groups [116].

The conjugation of phenolic hydroxyl groups usually decreases their radical scavenging capacity [92,93] but, depending on the position of this conjugation, both sulfate and glucuronide metabolites may present a much higher biological activity than the parental compound. This is the case, for example, of sulfate conjugates of daidzein [117] and glucuronides of urolithin A [118]. The presence of more hydroxyl groups, or sugar or sulphate moieties in the molecule, will not only affect the radical scavenging capacity of compounds but also other important properties for their antioxidant activity, namely their metal chelating capacity.

Despite their bioavailability controversies and their complex mechanism of action, oral phenolic antioxidant intake exhibits promising results as alternatives to drug interventions in preventing many chronic diseases, such as CVD and NDD, in at-risk populations. Dietary phenols, both in vitro and in vivo, have been demonstrated to display numerous beneficial bioactivities, namely showing anti-atherosclerotic, anti-hypertensive, and anti-cancer properties. Nevertheless, further studies are still needed for the complete understanding of the bioavailability and bioactivity of phenolic antioxidants in vivo, and to relate this bioactivity with their metabolites and the phenolic composition of foods.

## 4. Reactivity of Polyphenols: Radical Scavenging, Chelating Properties and Structure-Reactivity Relationships

### 4.1. Polyphenols as Free-Radical Scavengers

The effectiveness of phenolic compounds in inhibiting the production of ROS is related to their reactivity against radicals and to their effective concentrations at the reaction site. In this section, we will discuss the effects of phenolic architecture on their reactivity; meanwhile the concentration effects will be discussed later in Section 6. All polyphenols bear at least one -OH group attached to an aromatic ring, and mainly scavenge free radicals by H-atom transfer from the -OH group(s) of polyphenolics to the free radical, as seen in Scheme 1 (HAT mechanism), although other mechanisms (sequential proton-electron transfer (SPLET), proton-coupled electron transfer (PCET), and single electron transfer followed by proton transfer (SETPT)) may operate simultaneously, depending on the particular experimental conditions employed, as seen in Scheme 1 and Scheme 2 [42,119].

### 4.2. Structure-Reactivity Relationships

Several literature reports suggest that the oxidation–reduction, *E_p_*^0^, potential values of polyphenols correlate with their antioxidant activity, so that a decrease in the redox potential values leads to an increase in the radical scavenging ability [120,121,122,123]. Correlations between *E_p_*^0^ values and antioxidant activities have been proposed for some series of AOs, but data need to be taken with caution, because *E_p_*^0^ values are strongly affected by the nature and position of substituents on the aromatic rings of the polyphenols, altering strongly their antioxidant efficiencies [120,121]. Decreases in *E_p_*^0^ values have been reported for phenols that are methoxylated in the ortho-position of a phenolic hydroxyl group (e.g., ferulic acid series), and concomitant increases in their antioxidant activity have been observed. The introduction of a second methoxyl group in the other ortho position, with respect to the phenolic hydroxyl group (e.g., sinapic acid series), further decreases the *E_p_*^0^ value (relative to that of ferulic acid), usually increasing even more their antioxidant activity. Addition of a second hydroxyl group to the phenolic ring, both in the ortho or in the para position, e.g., catecholics, leads to an increase of the radical scavenging activity, which is not only due to the higher resonance stabilization of the phenoxyl radical intermediate, but also because the produced initial semiquinoid radical can be further oxidized to a quinone by the donation of a second hydrogen atom to another lipid radical with subsequent *ortho*- and *para*-quinone formation, respectively. In fact, monophenols have been described as oxidizing in a one-electron, one-proton irreversible step to a phenoxy radical [32], but the oxidation mechanism of AOs bearing the catecholic moiety usually involves a two-electron and two-proton reversible process [124]. *Ortho*-catechols are usually better antioxidants than most *para*-catechols, due to the stabilization of the semiquinone radical formed through strong internal hydrogen bonds [125].

An ethylenic side chain, connecting the aromatic ring to a carboxylic group, increases the reducing properties of phenolic acids. Nevertheless, the redox potential obtained for hydrocaffeic acid, where conjugation between the aromatic ring and the side alkyl chain is not possible, has been found to be lower than the one for caffeic acid. This suggests that the extension of the resonance over the alkyl side chain depresses the electronic density at the aromatic hydroxyl groups, decreasing their ability to donate H-atoms. These observations are in accordance with the bond dissociation enthalpy (BDE) and the ionization potential (IP) values that have been calculated for caffeic and dihydrocaffeic acid, that were found to be lower for the former. From the reactivity (kinetics) point of view [8], attachment of electron-donating substituents in the benzene ring of phenols, particularly at the *ortho* and/or *para* positions of the phenolic hydroxyl group, increases their radical scavenging activity by lowering the phenolic O–H bond dissociation enthalpy, thus increasing the rates of the reaction with peroxyl radicals.

### 4.3. Polyphenols as Chelators of Pro-Oxidant Metals

**Scheme 6:**
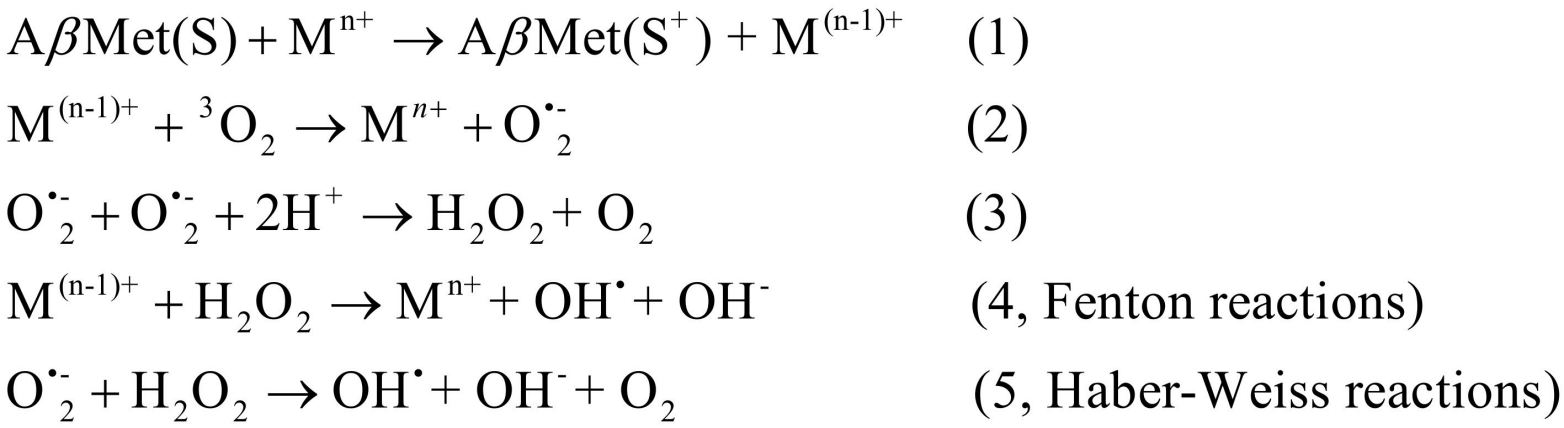
Examples of redox reactions involving ROS, metals (Mn^+^) and β-amyloid protein (Aβ) [132,133].

Transition metals play an important role as catalysts in the oxidation of biomolecules, when present in the appropriate oxidation state [8]. Most of the hydroxyl radical production in biological systems is a consequence of two main chemical reactions [126,127]. In the first one, the oxidation of biomolecules is initiated by the hydroxyl radical (HO^•^), generated in the reaction between the redox-active transition metal and hydrogen peroxide, by removing an electron from the participating metal ion (Fenton-type reactions) [126], as seen in Scheme 6. The original metal ions may be regenerated in a subsequent reaction (Mn^+^ + O_2_^•−^ → M(^(n−1)+^ + O_2_). This overall set of reactions constitutes the Haber–Weiss process, shown in Scheme 6, which is a combination of Fenton-type reactions and the reduction of Mn^+^ by O_2_^•−^, yielding M^(n−1)+^ and oxygen. Thus, control of metal levels in cells (especially Fe) is of particular importance, as increased concentrations may lead to undesirable reactions with oxygen to overproduce ROS [128,129,130,131].

**Scheme 7:**
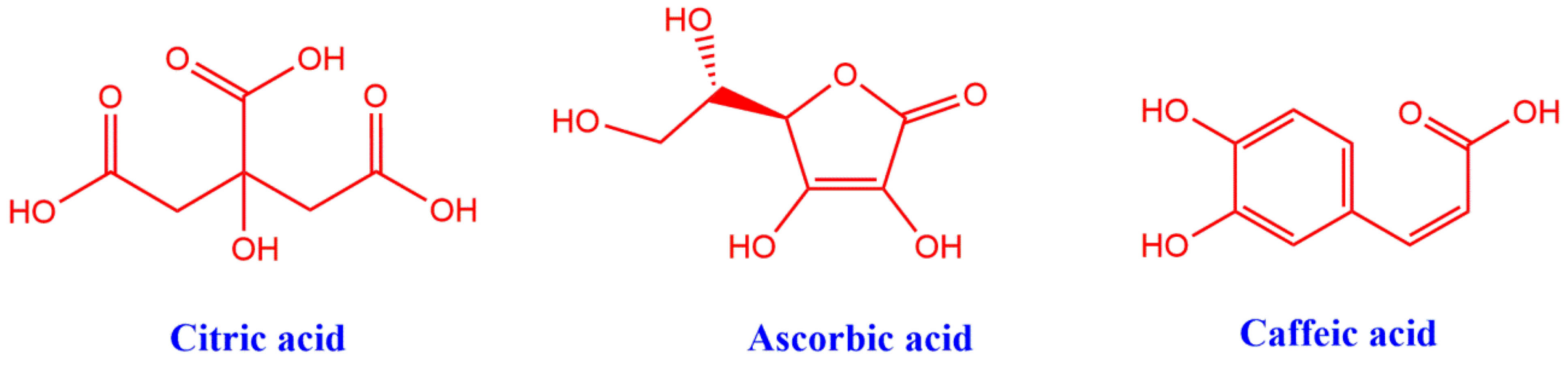
Some common metal chelators.

Antioxidants bearing only one -OH group in their structure (e.g., vanillic, syringic and ferulic acids) do not chelate metals, but those bearing the catechol or galloyl moieties, as seen in Scheme 7, are able to form complexes with typical stoichiometry of 3:1, 2:1 and 1:1 (antioxidant: metal) [134]. Flavonoids, as seen in Scheme 3, containing hydroxyl groups at the 3, 5, 3′, and 4′ positions, and carbonyl at the four position, possess three sites where metal complexes can form; the stoichiometry and stability depend on the number and type of the functional groups and pH [135].

## 5. Challenges on the Application of Antioxidants: Encapsulation and Delivery

There is a great interest in polyphenols as strong antioxidants, and their importance in food, pharmaceutical and cosmetic industries is evident. Although the antibacterial, antiviral, and anti-inflammatory potential of these antioxidants has been shown, the preparations containing polyphenols have limited in vivo activity. One of the most important factors that affect the delivery of polyphenols is their poor in vitro and in vivo stability [136,137]. Factors such as the temperature, light, and oxygen make them susceptible to degradation during storage and processing. Upon oral administration, the harsh gastrointestinal conditions, with an acidic pH and enzymes, lead to rapid degradation. The gastrointestinal barriers and poor solubility are also other factors responsible for low bioavailability. Another problem is the unpleasant taste of the polyphenols, which has to be masked before oral administration [138]. Therefore, in order to strengthen the potential usage of antioxidants, it is essential to maintain their structural integrity via special formulation strategies. Encapsulation, lyophilization, and emulsification are among these approaches [139].

### 5.1. Encapsulation Strategies for Antioxidants

Encapsulation has been a developing technology since the early 1930 [140]. It is the process of loading active molecules within carrier systems or shells, thus improving their physicochemical and biological properties [141]. The carrier material that protects the encapsulated material is generally made of polymers or lipids [18,50,142]. Natural (agar, albumin, gelatin, chitosan, starch, pectine, collagen, etc.) or synthetic polymers (polyethylene glycol, polyesters, poly vinyl pyrrolidone, poly vinyl alcohol, etc.) are commonly used. The major advantages these systems are as follows:

- Provide improved in vitro and in vivo stability of the encapsulated cargo,
- Modify the drug release profile and provide controlled release,
- Can be incorporated into different dosage forms including capsules, tablets, suspensions, gels, creams, etc.
- Site specific drug delivery,
- Reduce the inter- or intra- subject variability of the pharmacokinetic parameters,
- Good biocompatibility,
- Undesired side effects of the drugs can be reduced, and lower drug doses can be used which will improve patient compliance.

In order to provide the therapeutic use of polyphenols as new pharmaceutical products, their encapsulation is widely studied. The comprehensive advantages of encapsulating antioxidants include improved stability, controlled compound release, masked unpleasant odor or flavor, protection from the evaporation, improved solubility, permeability, and overall bioavailability. Besides that, encapsulation of polyphenols was shown to improve their potential cosmetic applications for skin pigmentation disorders, skin aging, and skin solar protection [143].

### 5.2. Antioxidant Loaded Drug Carrier Systems

In order to exert their intended effects, antioxidants must pass through many obstacles present in biological systems. Their encapsulation into micro- and nanocarriers has opened new horizons for improving their effects. Based on the production methods and materials, micro- or nanoparticles are obtained. The size of microparticles and nanoparticles ranges between 1–1000 μm and 1–1000 nm, respectively. The well-known structures are matrix and reservoir systems, and they can carry solid, liquid and gaseous materials [144]. These systems are characterized in terms of particle size and distribution, morphology, surface charge, encapsulation efficiency, production yield, in vitro drug release, stability, and toxicity [145]. The microcarriers usually have a better loading capacity when compared to nanocarriers, and the scale-up is easier [145]. For the microencapsulation of polyphenols, a diverse array of microparticular systems have been prepared by different manufacturing methods, including spray drying, lyophilization, extrusion, inclusion complexation, coacervation, and fluidized bed coating [146]. However, in recent years, nanosized drug delivery has received considerable interest, due to their numerous advantages. Due to their small size at nano range, and their increased surface area, nanocarriers have unique properties. They can circulate in the blood for a prolonged time, carry one or more active substances, target specific tissues, and improve the solubility and therapeutic efficacy of the encapsulated drug. Thus, besides reducing the toxicity of the active substance, its biocompatibility and bioavailability can also be increased. There are numerous studies emphasizing the success of polyphenol-encapsulated drug delivery systems and some of these are summarized in Table 5. Each of the listed carrier systems has their own advantages and disadvantages. The selection of the most suitable system for a specific antioxidant highly depends on its biological (absorption, distribution, metabolism, elimination, biological half-life, side effects, therapeutic index, etc.) and physicochemical (solubility, partition coefficient, pKa value, molecular weight, solubility, etc.) properties.

### 5.3. Importance of the Biointerface on Particulate Drug Delivery

Blood and plasma interactions, rapid clearance, presence of biological membranes, such as the blood brain barrier or gastrointestinal barrier, biological hydrogels (such as mucus), and the immune system reduce the effectiveness of drug carriers. The success of these systems highly depends on their bio interface, which can be tailored via physicochemical engineering. The morphological (particle size, shape, and rigidity) and topographical properties, surface chemistry, and the presence of responsive materials are several factors that influence the drug carrier interface [161].

Upon the absorption of plasma proteins onto nanocarriers, the protein corona structure is formed, and this highly affects their interactions with immune cells [162]. Surface morphology and chemistry are effective parameters on the protein corona formation. Particularly, the particle size of the carrier has been found to influence the thickness of the protein corona and the penetration of the encapsulated cargo through size-limited biological barriers [163]. Both the formulation (type and concentration of the chemical components, addition of ligands etc.) and process parameters (sonication time and rate, temperature, homogenization cycles and power etc.) can be changed to adjust the particle size.

It is important to consider the shape and rigidity of the particles as well when determining the in vivo efficacy of the carriers. Although the majority of the delivery systems are spherical, some other carrier shapes, such as disks, rods, and worms, are also reported. For instance, the phagocytic uptake of elongated particles was lower than that of spherical particles. and this was attributed to the alignment of the particles during the particle–cell interaction [164]. Non-spherical particles showed a higher circulation time than the spherical particles. The shape of the nanocarriers impacts the margination and adhesion process, and can alter the accumulation site of the carrier. This phenomenon can be used for site-specific drug delivery. Additionally, as the surface areas of non-spherical shapes are larger, surface modifications via ligands for targeted drug delivery is more feasible [165].

The surface chemistry of the particles is in direct contact with the biological medium. In order to increase the bio interface between biological membranes and the nano/micro particles, or to provide targeted drug delivery, the particle surface can be modified in terms of its charge, hydrophilicity, and ligand attachment. Besides, the modification of particles by dynamic materials that respond to biological stimuli, such as pH, redox, enzymes, and temperature improve their in vivo performance.

Therefore, the bio interface between the drug carrier and the biological system has an important role in the in vivo fate of the administered compound, and these carriers can be engineered accordingly at the formulation stage.

## 6. Some Common Delivery Systems

### 6.1. Cyclodextrins

Cyclodextrins, CDs, are a family of oligosaccharides, composed of six (α-CD), seven (β-CD), or eight (δ-CD) glucose residues, linked by a α-(1–4) glycosidic bond. In spite of α-,β-,δ-CDs being the most common CDs, a variety of CD derivatives have been developed with the objective of enhancing the physicochemical properties of natural CDs. For example, to increase the water solubility of β-CD (1.85% *w*/*v*, T = 25 °C [19], chemically modified β-CDs (hydroxypropyl (HP), randomly methylated (RM) β-CDs with aqueous solubilities > 600% *w*/*v* and >500, respectively, T = 25 °C [166] have been synthesized and widely used in the pharmaceutical and food industries. Due to their physical characteristics and chemical stability, (CDs) are widely employed as carrier systems to deliver the needed amount of bio-active compound to the targeted site for the necessary period of time [166,167,168].

**Figure 4:**
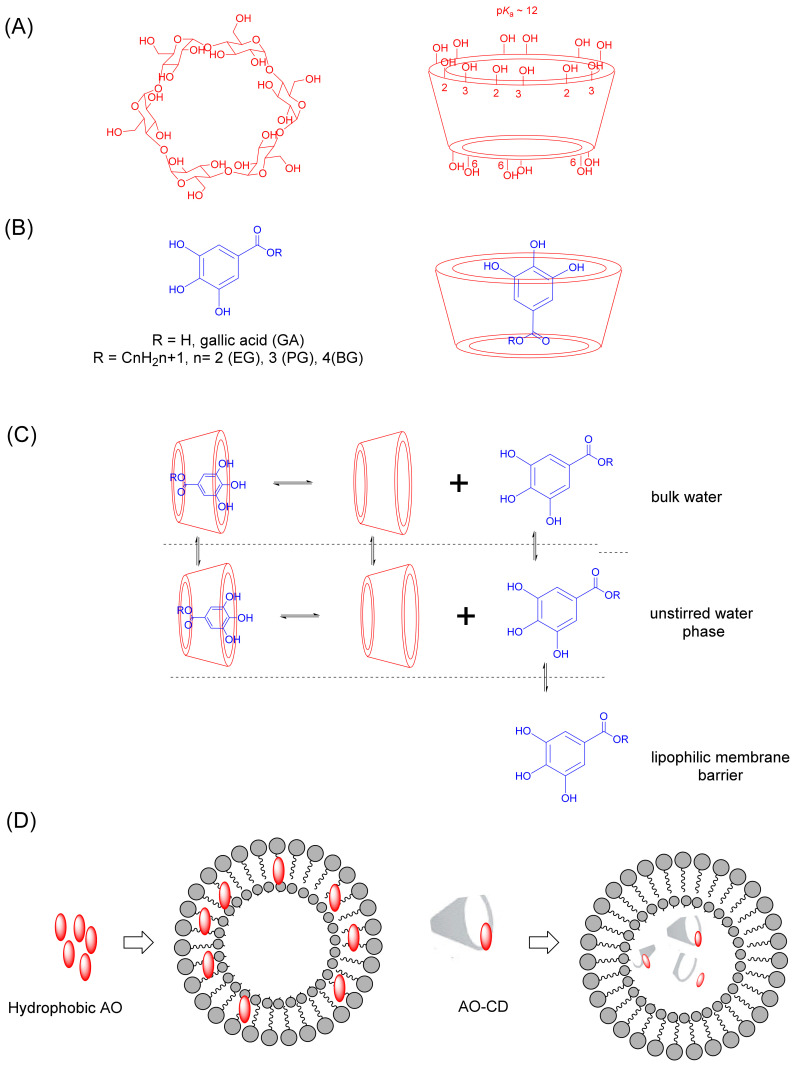
(**A**) Schematic representation of chemical structure of CDs, showing primary and secondary -OH groups and their conformation (truncate shape). (**B**) Inclusion complex for gallates (stoichiometry 1:1) with the phenolic ring inserted into the CD cavity and the -OH groups pointing outwards. (**C**) Representation of potential distributions of a free hydrophilic CD, gallates, and a gallates–CD complex between bulk water, unstirred water, and membrane. Gallates–CD complex formation can enchance gallates diffusion through the unstirred water phase and, consequently, their membrane permeation (adapted from reference [169]. (**D**) Schematic representation of potential localization of a free AO and AO − CD complex in liposomes.

CDs are commonly described as a truncated cone or torus, with a hydrophobic inner cavity and a hydrophilic external surface, as seen in Figure 4A. The nonbonding electron pairs of the glycosidic oxygen bridges are directed toward the zone inside, leading to a high electron density. In contrast, the primary and the secondary hydroxyl groups are distributed on the narrow (primary face) and wider (secondary face) rims, respectively, of the truncated cone shape.

This structure enables them to form host–guest complexes with a variety of polar and apolar antioxidants (“guest“ or substrate, AO), as seen in Figure 4B, changing their physical-chemical properties (e.g., solubility, antioxidant efficiency). CD–antioxidant (AO − CD) inclusion complex formation, characterized by an inclusion constant K_AO_ (Equation (1) for a 1:1 complex), is a dynamic equilibrium process where AO − CD is formed by the replacement of the included water molecules by the less polar substrates.

(1) $$\mathrm{AO}+\mathrm{CD}\overset{k_{F}}{\underset{k_{D}}{⇌}}\mathrm{AO}-\mathrm{CD}K_{\mathrm{AO}}=\frac{k_{F}}{k_{D}}=\frac{\left[\mathrm{AO}-\mathrm{CD}\right]}{\left[\mathrm{AO}][\mathrm{CD}\right]}$$

The main forces involved between the substrate (AO) and CD are weak, non-ionic interactions (hydrophobic, electrostatic, van der Waals forces, hydrogen bonding, charge transfer interactions etc.) but never by complete covalent bonds. These weak interactions are responsible for keeping the guest substrate and host together, with a complete or partial adjustment of the cavity [168,170]. Commonly, the stoichiometry of the inclusion complex AO − CD is 1:1, representing the interaction of a single AO molecule with the CD; however, other stoichiometries (1:2, 2:1, 2:2) can also be found [168]. Parameters such as the relative sizes and shapes of the free molecules can affect to the stoichiometry of the inclusion AO − CD complex.

#### 6.1.1. Cyclodextrin Complexation with Antioxidants

CDs can encapsulate poorly water-soluble polyphenols, not only to improve their solubility, but also to protect them from side-effects of their environmental conditions (light, temperature, pH, etc.). The formation of inclusion complexes with CDs has been observed with a variety of antioxidants, including rosmarinic acid [171,172], ferulic acid [173], and gallic acid [15,174], as well as resveratrol [175], quercetin [176], tea polyphenols, [177] and phenol-rich plant extracts [15], among others. Here, the complexation of antioxidants with CDs leads to enhanced water solubility of free compounds, as well as increased stability, bioavailability, and in vitro antioxidant activity. In this sense, García-Pérez et al. [178] showed that the value of the concentration of gallates required to reduce the DPPH^•^ radical concentration by 50% (%Inhibition = 50%, EC_50_ value) decreased by ~1.3–1.5 fold when they were encapsulated in CDs, Table 6. Yallapu et al. [179] prepared a β-CD based curcumin drug delivery carrier and showed that β-CD-curcumin enhanced the distribution of curcumin in prostate cancer cells when compared to free curcumin, increasing its therapeutic activity.

CDs can modify AO’s permeability through biological membranes under certain medium conditions, when the contribution of an unstirred aqueous phase to the overall barrier function of a membrane is significant and it becomes the rate limiting step in the absorption process for a poorly water soluble AO [180]. So, hydrophilic AO − CD complex formation can regulate AO flux though membranes, based on their interactions with the unstirred water phase that separates the bulk media from biological membranes, including the cornea, reproductive tract, and the gastric mucosa, as seen in Figure 4C [180]. On the other hand, observations also show that hydrophobic AO − CDs (e.g., methylated CDs) enhance AO flux by changing membrane (e.g., in the nasal mucosa) properties [167]. CDs can form complexes with membrane components such as phospholipids, cholesterol, or other lipophilic molecules, producing changes at a cellular level or within biological barriers [167,170]. Besides, CD inclusion complexes can improve absorption by passing p-glycoprotein (P-gp)-mediated efflux activity. P-gp is a transporter expressed in the intestines, liver, kidney, etc., that promotes the efflux of guest molecules, which constitutes a major absorption barrier [167,170]. These properties make them a helpful tool for the development of new drug delivery systems.

#### 6.1.2. Cyclodextrin-Based Nanocarriers of Antioxidants

The combination of CD complexation with nanotechnology represents a potential approach regarding the development of multifunctional CD-based delivery nanocarriers to address therapeutic needs [167]. So, the development of cyclodextrin-based compounds will likely be accelerated in the coming years, and involves the combination of two approaches in a single nanocarrier: (1) the formation of AO − CD complexes and (2) the encapsulation of complexed AO into a delivery system.

The currently used cyclodextrin-based nanocarrier of AOs can enhance their bioavailability, extend the existence of AO in systemic circulation, decrease its toxicity, and has a proven controlled release, addressing some drawbacks linked with each individual system (that can reduce the CD’s effectiveness, as delivery systems have a fast elimination from the bloodstream after their in vivo administration, or a potential substitution of the encapsulated AO by other molecules with higher affinity for the inner CD cavity may occur) [181]. Some of the cyclodextrin-based carriers of AOs include liposomes, nioxomes, nanosponges, micelles, polymeric millirods, and magnetic nanoparticles, among others [181]. For example, liposomes in the presence of CDs have demonstrated to be a promising delivery system for enhancing the entrapment activity and stability of antioxidants such as curcumin, as seen in Figure 4D. CD–resveratrol nanoparticles showed an improved therapeutic efficiency when compared to free resveratrol by passive targeting, while maintaining its antioxidant effects [182]. CDs are also known to enhance quercetin’s permeation through biological membranes. In this way, to increase quercetin’s brain uptake, nasal powders composed of quercetin-cyclodextrin lyophilizates, mixed with spray-dried microparticles of mannitol/lecithin, were formulated, increasing their solubility 19–35 times when complexed with cyclodextrins [183]. In this way, the employment of AO delivery systems has focused on the combination of colloidal vesicles and CDs, constituting a promising strategy to find an effective therapy with the least possible side effects.

### 6.2. Microemulsions

Microemulsions are self-assembled colloidal systems that are formulated from oil, water, a surfactant, and a cosurfactant. They are thermodynamically stable and transparent mixtures, with droplet sizes between 10 and 100 nm [184]. In microemulsions, the stabilization of the droplets is provided by the reduction of the interfacial tension between the two phases via the surfactant and cosurfactant.

Microemulsions are one of the most preferable delivery systems because of the simplicity of their preparation process. Hydrophilic and lipophilic substances can be loaded in different phases of microemulsions with a high encapsulation efficiency, and they can be administered as liquid forms. They provide a controlled release and improve the stability of encapsulated cargo against hydrolysis and oxidation [185]. It is known that microemulsions resist gravitational collapse due to their small droplet size. Various factors affect the droplet size in microemulsion systems, including the alkyl chain length of the oil, the surfactant type, and the rigidity of the interfacial film. The nanodroplet size improves the absorption and bioavailability of the substances by increasing the surface area to volume ratio [186]. Different classes of surfactants are used to prepare microemulsions. The uncharged structure, stability against pH changes, and safety profile make non-ionic surfactants (such as Tweens, Spans, Brij, Pluronics, etc.) more advantageous. The cosurfactants (such as alcohols, glycerol derivatives, polyglycerols, etc.) are used to further reduce the interfacial tension and provide flexibility to the interfacial film. The assembly of microemulsions is possible because the interfacial tension between the immiscible phases reach close to zero. This is explained by several approaches, including film theory, solubilization theory, and thermodynamic theory [187]. The interfacial free energy should be reduced to facilitate the formation of microemulsions [188]. The thermodynamics of microemulsions can be explained by the Equation (2), where $DG_{f}$ stands for the free energy of formation, γ: Surface tension of the oil–water interface; DA: The change in the interfacial area on microemulsification, DS: The change in entropy of the system, and T: Temperature.

(2) $$DG_{f}=\gamma DA-TDS$$

**Figure 5:**
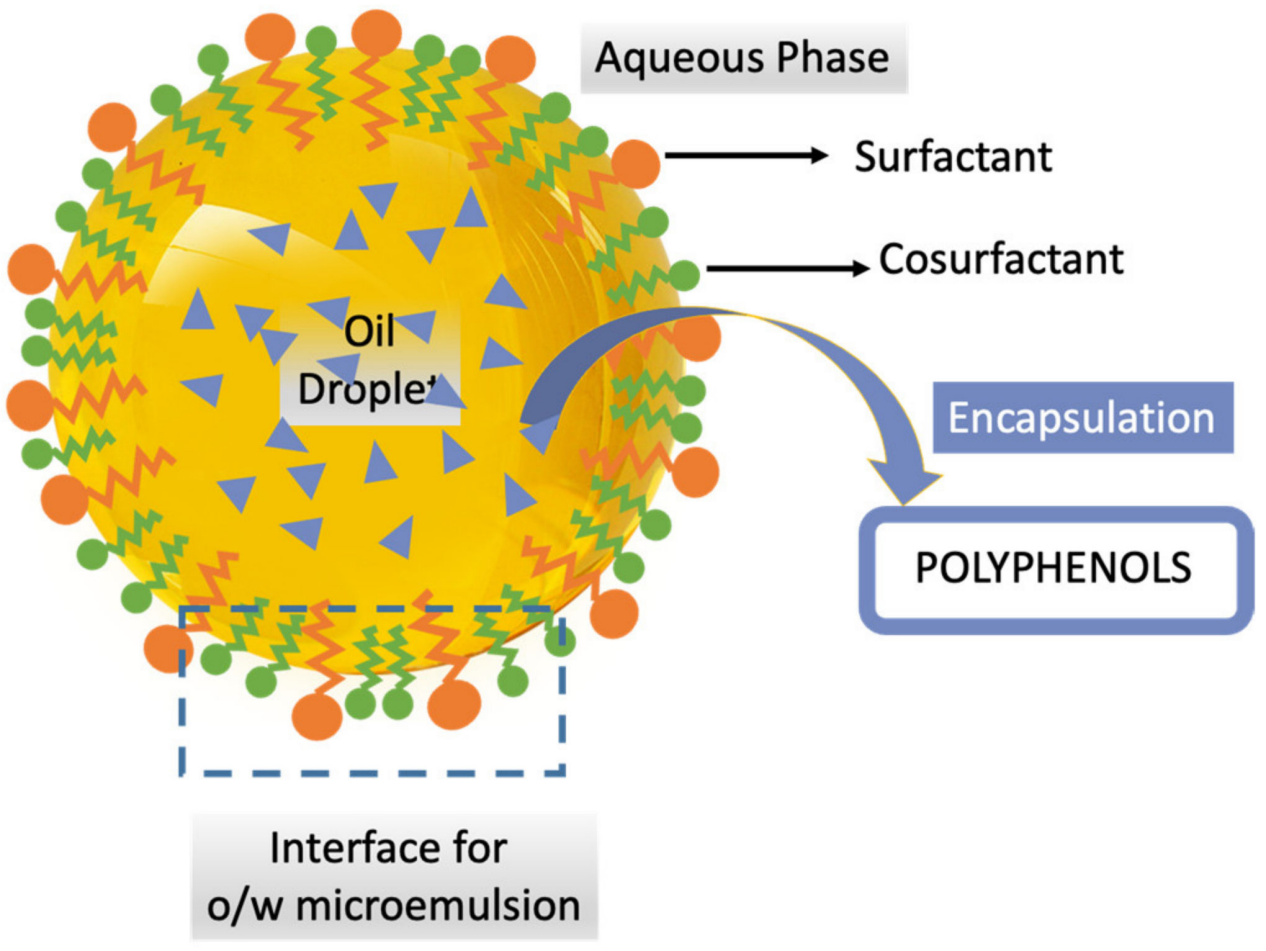
Schematic representation of the droplet structure of an oil-in-water microemulsion carrying polyphenolic agents.

The phase titration and phase inversion are the main microemulsion production methods [189]. Microemulsion formulations (Figure 5) are designed to deliver polyphenols by a wide range of administration routes, including oral, parenteral, transdermal, nasal, etc. [187].

Encapsulation of curcumin into O/W and W/O/W microemulsions (droplet size 10–20 nm) improved its solubility, stability, and antioxidant capacity. The position of curcumin within the inner structure of microemulsions was confirmed by ^1^H NMR studies [190]. Improved oral bioavailability (~2.5 times higher) was obtained after administration of curcumin-loaded turmeric oil microemulsions, with a droplet size of 29 nm in zebra fish. Besides, ex vivo drug permeation studies in the chicken gut sac also revealed the enhancement of flux when compared to curcumin solution [191]. The hepatoprotective activity of polyphenols-enriched fraction (Fr 1), extracted from Ajwa fruits, was shown in cell culture and in vivo mice models. Fr 1-carrying microemulsions significantly improved the oral bioavailability of the compound when compared to the suspension form [192]. The solubility of quercetin was improved by microemulsions and the in situ studies revealed an increased absorption percentage in different regions of rat intestine [193]. Overall studies indicate that microemulsions can increase the oral bioavailability of compounds by paracellular diffusion and lymphatic transport. They also contribute to the stability of molecules by protecting against enzymatic degradation in the gastrointestinal tract [46,47].

Gallic acid microemulsions were shown to improve the antioxidant activity of the compound [194]. Incorporation of chitosan to microemulsions further improved the transport of gallic acid through RPMI 2650 cell lines (human nasal epithelial cell line) without exhibiting any cytotoxic effects. Thus, this formulation was found promising for the nasal delivery of antioxidants as an alternative to the oral route [195]. Brain-targeted delivery of curcumin via the nasal route was successfully shown via microemulsion-based gel formulations containing polycarbophil as a mucoadhesive agent [196].

Several factors potentialize the drug delivery to skin via microemulsions. The low interfacial tension between the water and oil phases is thought to improve the contact to skin. The excipients of microemulsions also act as penetration enhancers [197]. Topical microemulsion formulations of catechin have improved the cumulative amount of the transported drug through Sprag Dawley skin layers up to 111.6 fold, and shorten lag times in in vitro experiments [198]. In vitro and in vivo skin penetration of quercetin was increased via canola oil-based w/o microemulsions, and this was found to be effective against UVB damage [199]. Efficient delivery of resveratrol to the dermis layer of the Yucatan micropig (YMP) skin was obtained using an oil-in-water (o/w)-type microemulsion containing sucrose laurate as a surfactant [200].

As mentioned, the superiority of polyphenol-containing microemulsions has been demonstrated by various in vivo and in vitro studies. However, some considerations should be taken into account when using these systems. As high concentrations of surfactants and co-surfactants are required to stabilize the nanosized droplets of microemulsions, they should be investigated in terms of their potential toxicity. Environmental parameters, such as temperature and pH, may lead to stability problems such as “breaking” and “phase separation” [40].

### 6.3. Emulsions and Nanoemulsions

**Scheme 8:**
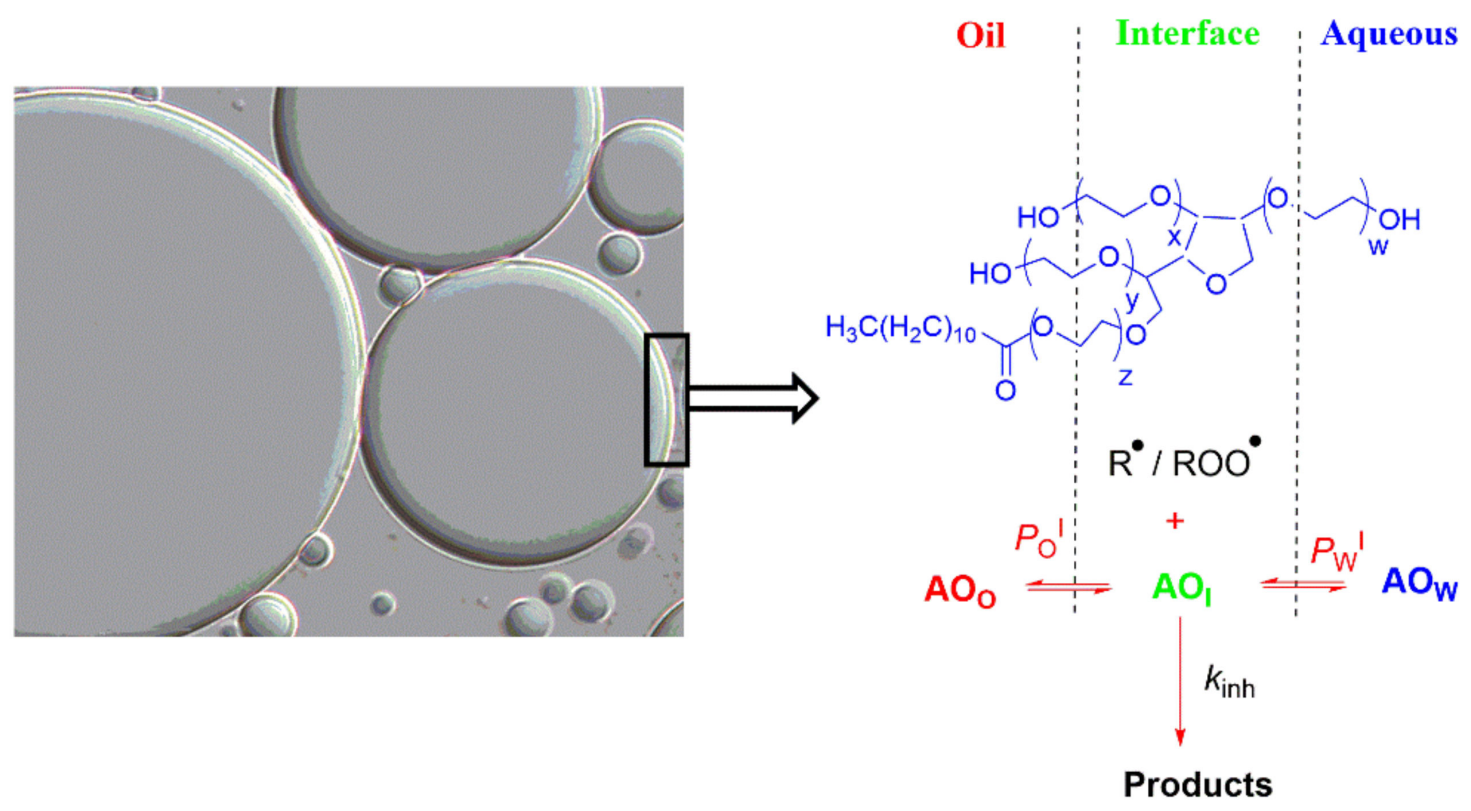
Oil-in-water emulsions and nanoemulsions contain three-dimensional regions (oil, interfacial, and aqueous) where antioxidants can be located. A small portion of the regions is displayed in the right picture, showing the chemical structure of a surfactant and the potential locations of an antioxidant. The interfacial region is the main reaction site where the inhibition reaction between antioxidants and lipid radicals takes place [39,178].

Emulsions and nanoemulsions are thermodynamically unstable mixtures of two immiscible liquids (oil and water), one dispersed as droplets throughout the other one (continuous phase), that can be stabilized kinetically by the addition of surfactants and co-surfactants that form an interfacial region, separating the oil from the aqueous solution, as seen in Scheme 8 [201]. The main difference between nanoemulsions and emulsions is the size of the droplets in the system. Although there is not a clear separation, nanoemulsions are considered to have droplets with diameters lower than 200 nm. Nevertheless, nanoemulsions have some physicochemical properties that are distinct from those of emulsions: they can be optically clear, due to the limited light scattering by small droplets; they can be highly stable against gravitational separation, due to weak gravitational forces and Brownian motion effects, and they may present higher bioavailability.

When compared with emulsions, the emulsifier concentration used is usually critical, and plays a role in stabilizing droplets through both repulsive electrostatic forces and steric hindrance. Although surfactants are usually used as emulsifiers, both proteins and lipids can also be used in the stabilization of emulsions and nanoemulsions [58].

**Figure 6:**
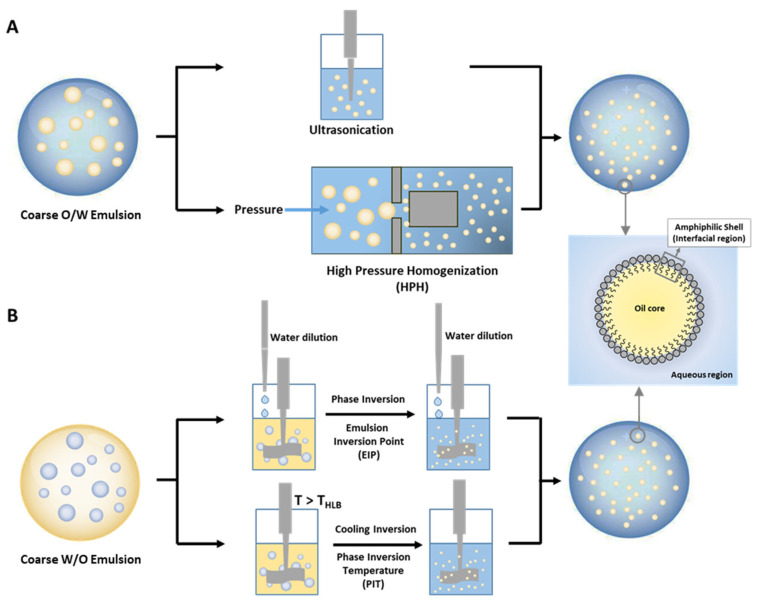
Common methods for preparing oil-in-water nanoemulsions. (**A**) The high energy methods, such as high pressure homogenization (HPH) and ultrasonication, break coarse emulsions drops into smaller droplets. (**B**) The low-energy methods start with W/O coarse emulsions, and droplets are broken into smaller droplets during the low interfacial tension state that occurs during phase transition, induced by the water dilution. In the phase inversion temperature (PIT) method, the phase inversion occurs by cooling the mixture. W/O nanoemulsions can be prepared by employing the same methodology.

Nanoemulsion preparation can be achieved basically by two methodologies, as seen in Figure 6: by high-energy and by low-energy methods [1]. High-energy methods, namely ultrasonication, high pressure homogenization (HPH), evaporative ripening [202], and microfluidization, usually consume a significant amount of energy and require multiple passes/steps in order to obtain the required droplet size [201]. In contrast, low-energy methods, such as phase inversion temperature (PIT) [203], emulsion inversion point (EIP) [204], and bubble bursting at the oil/water interface [205], use specific material properties to make small droplets without consuming a significant amount of energy.

Nanoemulsions have a range of application fields, including hydrophobic drug delivery [202], in the bioaccessibility and digestibility improvement of some nutrients in the gastrointestinal tract and skin, and can also be used in the preparation of more complex particles, such as compartmentalized nanoparticles [202].

The physicochemical characteristics of a nanoemulsion are largely dependent on the composition, physical state, size, aggregation state, electric charge, and interfacial composition of droplets. In turn, the final droplet size can be influenced by the relative viscosity (μd/μc) of the oil and aquouse phases, the emulsifier concentration ([S]), and the emulsifier properties (thickness, charge, hydrophobicity, length, etc.) [206]. Moreover, the droplet electric characteristics are largely determined by the nature of the surface-active substances adsorbed to their surface, including the emusifier charge and the ionic composition of the aquouese phase [206], which play a major role in determining the physical and chemical stability of the nanoemulsion, its bioavalability, and the possibility of it being used in the production of compartmentalized nanoparticles. The knowlege of these parameteres permits us to tailor the properties of the nanoemulsion droplets, in order to produce the required physicochemical or physiological properties for a specific application. Typically, nanoemulsions contain a range of different droplet sizes, and therefore, their dimensions are characterized in terms of size distribution, mean droplet diameter, and polydispersity index. In contrast with conventional emulsions, in nanoemulsions, the interfacial region can achieve an important droplet volume [207], as can be envisaged from Equation (3), where Ø*_S_* (=*V_S_*/*V_C_*_+*S*_) is the volume of the emulsifier (*s*) divided by the effective volume of the overall droplet (=core + *s*), δ*_S_* is the thickness of the emulsifier, and *r* is the radius of the oil droplet (core). Consequently, the choice of the best emulsifier is extremely important in the development of a nanoemulsion for a particular application.

(3) $${Ø}_{S}=\frac{{\left(r+\delta_{S}\right)}^{3}-r^{3}}{{\left(r+\delta_{S}\right)}^{3}}\pi r^{2}$$

The properties, formation and stability of nanoemulsions also depend on the bulk physicochemical properties of the oil phase, namely their hydrosolubility, interfacial tension, viscosity, density, phase behavior, and chemical stability [207,208]. These properties often limit the methodology that is able to be used in their prepration. For example, low γ usually facilitates droplet formation both in phase-inversion and spontaneous emulsification, as well as in high-energy methods, but it may lead to poor emulsifier affinity for droplet surfaces, promoting droplet coalescence.

In the food industry, nanoemulsions are usually prepared using edible oils, due to their low cost, and their functional and nutritional caracheteristics, such as olive, corn, soybean, sunflower, flaxseed, and more recently, fish and algae oils. The major constituints of these oils are long-chain triacylglycerols and, therefore, the preparation of nanoemulsions with these oils is often difficult by the phase-inversion temperature (PIT) method, because of their very high hydrophobicity, as well as by high-pressure homogenization methods, because of their high viscosity [201,208]. However, once a nanoemulsion has been prepared they are often highly physically stable. In contrast, other less common edible oils, such as flavor and essential oils, with a relatively high polarity, low interfacial tension, and low viscosity, can easily produce very small droplets by both methodologies but, once produced, these nanoemulsions have a low stability because, due to their higher hydrosolubility and lower interfacial tension, they suffer from Ostwald ripening or coalescence. Several strategies can be used to prevent the low stability of nanoemulsions, and the interested reader is referred to some reports [201,209]. Low-energy methodologies’ limitations also include the impossibility of using proteins or polysaccharides as emulsifiers and the need of a relatively high concentration of synthetic surfactants, which may hinder their application in many foods.

Nanoemulsions can be used to design functional foods with ingredients that are difficult to incorporate, due to low water solubility. Lipophilic components are usually mixed with the oil phase prior to nanoemulsion preparation, so that they end up trapped within the oil core of droplets. For example, the bioaccessibility of the highly lipophilic molecule of β-carotene, a pigment with important antioxidant and health benefits, can be improved by being incorporated [207,210,211] in nanoemulsions prepared by different methods and with different compounds, such as β-lactoglobulin, which is used as a biocompatible emulsifier. Another antioxidant that is quite often incorporated into nanoemulsions because of its easier digestion and higher bioassessibility is curcumin, an anti-inflammatory agent [212]. Oil-soluble vitamins [201] have also been incapsulated in nanoemulsions.

Consumption of fish oil rich in omega-3 polyunsaturated lipids and oil-soluble vitamins is linked to improved human health, which has partly been attributed to their important role in brain and cardiovascular health [206]. However, the use of this oil is a major challenge, due to its low hydrosolubility, unpleasant fishy taste, bioavailability, and its high susceptibility to oxidation, due to the high degree of lipid unsaturation. Emulsified systems have a high potential for overcoming some of these challenges since they can be designed to have good kinetic stability, oral bioavailability, to mask undesirable flavors [206], and can be used not only in parenteral nutrition but also to fortify beverages, sauces, and desserts, or to increase the bioavalability of omega-3-FA formulations. The lower oxidative stability of these preparations has hindered their application and safety. Efforts to minimize the impact of omega-3 FA oxidation have increased in the last few years by optimizing food processing, packaging and refrigeration [206]. However, controlled storage can be expensive and time consuming. Thus, for economical and practical reasons, the addition of AOs is one of the best strategies that is employed in industry to retard lipid oxidation.

One of the increasing areas of nanoemulsion applications is in dermatological products, both in the treatment of skin pathologies and as anti-aging skincare, due to the effective penetration of nano droplets onto the skin’s surface, their pleasant visual appearance, and their stabilization of ingredients, including vitamins and antioxidants [201,213,214]. Because of its tunable droplet size, nanoemulsions reported less skin irritation, due to the penetration of droplets over skin via hair follicles and pores and not affecting healthy tissues [69,70,71]. There are several examples of nanoemulsion formulations with high bioactive transdermal availability without degradation using flavanones, curcumin, resveratrol, quercetin, narigenin, and retinyl palmitate with efficient antioxidant and anti-aging activities [214,215,216].

The physical location of antioxidants within nanoemulsions depends on their molecular properties and solubility in the oil, aqueous, and interfacial region. Studies concerning the location of antioxidants in emulsified systems has led to the important conclusion that droplet size has a negligible effect on the distribution and effective concentration of antioxidants (and probably of any other molecule) in the aqueous and interfacial regions of the emulsion, as the partition constants of the molecules between the aqueous and interfacial regions and between the oil phase and interfacial region do not change with the size of the droplet [217,218,219]. Further details on the distribution of antioxidants in emulsions and nanoemulsions are provided in the next section.

### 6.4. Antioxidant Distributions and Efficiencies in Emulsions and Nanoemulsions

Polyphenolic antioxidants and antimicrobials are added in different forms to control lipid oxidation and the growth of pathogenic and spoilage microorganisms [220]. Emulsions and nanoemulsions show great promise as antioxidant carriers in the nanoscale dimension [18,50,221]. Their advantages include, among others, an improvement in the protection of the delivery system against oxidation of lipidic components, antimicrobial action by improving intracellular penetration, and the delivery of required drugs to specific sites by preparing tailored surfaces of the carriers, for instance, changing the nature of the oils and of the emulsifiers employed to stabilize kinetically the nanoemulsions [18,50,221,222].

Fundamental questions that arise when using delivery systems include how the polyphenols are distributed between the oil, water, and interfacial regions of the emulsions and what are the real concentrations in each region. Linked to these basic questions, others also arise concerning the effects of the environmental (e.g., acidity, temperature) and formulation (type of oil, oil-to-water ratio, the surfactant employed etc.) conditions on their partitioning and their efficiency. Indeed, the interfacial oil–water film plays a key role because its solvent properties are different from those the bulk water and oil, and antioxidants may accumulate in this region, affecting their efficiency [218,219,223,224,225,226].

Emulsions are mixtures of oil, water, and surfactant where either the water or the oil can act as dispersing phase, leading to the formation of oil-in-water and water-in-oil emulsions, respectively. Scheme 8 shows the droplets of an oil-in-water emulsion and the conceptual division in three distinctive regions: the droplet core (oil), the continuous aqueous phase, and the three-dimensional interfacial region. The interfacial region (composed of surface-active molecules, oil, water, and other components) is highly anisotropic and comprises a narrow region (typically 2 to 20 nm thick), enclosing the oily droplet core [18,50,227].

Studies on the effects of factors controlling the allocation of antioxidants in emulsions (e.g., oil-to-water ratio, acidity, temperature, type of oil) indicate that the surfactant volume fraction is the main parameter controlling their distribution, except the acidity of the solution for phenolic acids or vitamin C. Results on the distribution of antioxidants in emulsions show, therefore, that: (i) the fraction of AOs in the interfacial regions of the emulsions do not correlate directly with the hydrophobicity of the AO; and (ii) that the fraction of AO in the interfacial region reaches a maximum for AO’s of intermediate polarity or intermediate alkyl chain length. An optimal balance of hydrophobicity and hydrophilicity (headgroup polarity) is required for maximum solubility of the AO in the interfacial region of the emulsion and, therefore, maximum antioxidant efficiency. Hence, the antioxidant and antibacterial properties of the interfacial region can be improved by targeting antioxidants to the interfaces of emulsions, where their local, effective, concentration is much higher than the stoichiometric one [18,50,227].

Further research is, however, needed to achieve a better understanding on the location and orientations of polyphenols in the interfaces of emulsion-based delivery systems. This definitely will allow for an optimal control of the subsequent release of incorporated bioactives in specific sites, allowing for the preparation of target-tailored systems and increasing their efficiency in the biology, medical, pharmaceutical, and cosmetic areas. Certainly, a deeper knowledge on the location and distribution of the bioactives in the system will aid in the development of new delivery systems, not only as nutritional supplements, but also with potential benefits to human health [8,15,47].

## 7. Concluding Remarks

This review aims to provide a general overview of the biological activity of polyphenolic antioxidants, and strategies to improve their bioavailability and thus, their efficiency, to reduce or inhibit ROS damage. Some, but not all, of their therapeutic effects of polyphenolic antioxidants are known but further both in vitro and in vivo (clinical) studies will help to evaluate and fully understand their bioavailability, bioactivity, and to develop new antioxidant strategies, including their delivery to target areas, in attempting to prevent oxidative stress, minimizing damage to DNA, protein, cells, and other important molecules and tissues, while maintaining the cellular redox homeostasis.

Oxidative stress is of key importance in the pathogenesis of many health diseases and the use of exogenous antioxidants to improve the endogenous antioxidant system could be used to reduce it. While there is a large body of research demonstrating the general effect of oxidative stress on signaling pathways, little is known about the effects of AOs on the cellular regulation of ROS, their bioavailability, and their bioactivity at the reaction sites. Antioxidants need to be properly positioned (location and orientation) in compartmentalized systems to react efficiently with excesses of ROS and to protect biomolecules from oxidative damage. In these systems, the biointerfaces are most probably the critical areas where the inhibition reaction takes place, and studies about the delivering and partitioning of antioxidants are critical to fully understand and optimize their performance. Certainly, a better understanding of the mechanisms through which antioxidants act, and where and when they are effective, may give a more rational strategy for extending food shelf-life and the prevention of health diseases, and so will help to solve several limitations that challenge the ability to therapeutically apply antioxidant strategies.

Dispensation of bioactives to humans is challenging because their delivery and release into the surrounding medium needs to be controlled to improve their bioavailability. In addition, the actual bioactive may need to be protected from the environment to maintain its therapeutic properties. Research strategies and directions into the administration and release of bioactives are mostly inspired by the modeling of biological systems. For example, a nice model that inspired many researchers is based on the protective role of cell walls, controlling the exchange of matter with the surrounding medium and sheltering the interior components from the extracellular environment. Based on these observations, cyclodextrins and colloidal systems such as emulsions, nanoemulsions, and microemulsions have a great potential as delivery systems and, for example, are widely employed in parenteral nutrition as energy sources and, at the same time, for the delivery of multiple nutrients.

In the last few years, lipid-based delivery systems have gained much attraction because they have a great potential in parenteral nutrition as energy sources and, at the same time, for the delivery of multiple nutrients. Research on the encapsulation of antioxidants has attracted great interest in the functional food, nutraceutical, and pharmaceutical industries, due to their health benefits to humans. Certainly, the use of encapsulated polyphenols, instead of free compounds, can effectively overcome solubility problems and, at the same time, preserve the stability, bioactivity, and bioavailability of the polyphenol. It appears, therefore, of outmost importance to control the distribution and partitioning of the bioactives (e.g., antioxidants), nevertheless, knowledge on how partitioning influences their efficiency is challenging. Predicting bioactive distributions and gaining some control on their partitioning is necessary for developing specific strategies to encapsulate and deliver antioxidants. 

## Figures and Tables

**Table 1:** Main radical and non-radical reactive oxygen and nitrogen species (ROS and RNS, respectively).

ROS		RNS	
Free Radicals	Non-Radicals	Free Radicals	Non-Radicals
Hydroxyl (HO^•^)	Hydrogen peroxide (H_2_O_2_)	Nitric Oxide (NO^•^)	Dinitrogen trioxide (N_2_O_3_)
Superoxide anion (O_2_^•−^)	Singlet oxygen (^1^O_2_)	Nitrogen dioxide (NO_2_^•^)	Dinitrogen tetraoxide (N_2_O_4_)
Lipid peroxyl (ROO^•^)	Ozone (O_3_)		Peroxynitrite (ONOO^−^)
Thiyl (^•^RS)	Lipid peroxide (ROOH)		Nitrosothiols (SNOs)

**Table 2:** Half-lives and typical rate constant kexp values of some ROS. L = linolenic acid. Data from ref. [8].

Radical	*k*_exp_ (M^−1^ s^−1^)	t_1/2_ (s)
HO^•^	10^9^–10^10^	10^−9^
RO^•^	10^6^–10^8^	10^−6^
ROO^•^	10^1^–10^3^	10
L^•^	10^4^–10^8^	10^−8^
O_2_^•−^	1	-
HOO^•^	10^0^–10^3^	-
^1^O_2_		10^−6^

**Table 3:** Values of the rate constants for the reaction of ROS with some fatty acids and amino acids. Data from refs. [12,13,14].

Rate Constant Values (M^−1^ s^−1^) for Reactions of ROS with Fatty Acids (H-Abstraction)				
	Oleic	Linoleic	Linolenic	
HO^•^	~10^9^	9 × 10^9^	7.3 × 10^9^	
RO^•^	3.3 × 10^6^	8.8 × 10^6^	1.3 × 10^7^	
ROO^•^	1.1	60	120	
O_2_^•−^	~0	~0	<1	
HOO^•^	~0	1.1 × 10^3^	1.7 × 10^3^	
^1^O_2_	7.5 × 10^2^	9.7 × 10^3^	1.2 × 10^4^	
**Rate Constant Values (M^−1^ s^−1^) for the Reaction of ROS with Aminoacids**				
	**Histidine**	**Tyrosine**	**Cysteine**	**Methionine**
HO^•^	4.8 × 10^9^	1.3 × 10^10^	1.9 × 10^10^	7.4 × 10^9^
O_2_^•−^	~1	~24	~0.4	~0.33
^1^O_2_	4.6 × 10^7^	1.3 × 10^7^	5.0 × 10^7^	1.3 × 10^7^

**Table 4:** Major enzymatic ROS scavengers in the antioxidant mechanisms. RX = redoxin, DHA = dehydroascorbate, AsA = ascorbate, MDHA = monodehydroascorbate. Adapted from ref. [8].

Scavenger	Acronym	Catalyzed Reaction
Superoxide dismutase	SOD	$\begin{array}{l} M^{(n+1)+}{+\mathrm{SOD}+O}_{2}^{-}\to M^{n+}{+\mathrm{SOD}+O}_{2}\\ M^{n+}{+\mathrm{SOD}+O}_{2}^{-}{+2H}^{+}\to M^{(n+1)+}{+\mathrm{SOD}+H}_{2}O_{2} \end{array}$
Catalase	CAT	${2H}_{2}O_{2}\to O_{2}{+2H}_{2}O$
Ascorbate peroxidase	APX	$H_{2}O_{2}+\mathrm{AsA}\to {\mathrm{DHA}+2H}_{2}O$
Monodehydroascorbate reductase	MDHAR	$\mathrm{MDHA}+\mathrm{NAD}(P)H\to {\mathrm{AsA}+\mathrm{NAD}(P)}^{+}$
Glutatione peroxidase	GPx	$\begin{array}{l} {2\mathrm{GSH}+H}_{2}O\to {\mathrm{GSSG}+2H}_{2}O\\ 2\mathrm{GSH}+\mathrm{ROOH}\to {\mathrm{GSSG}+\mathrm{ROH}+H}_{2}O \end{array}$
Glutatione transferase	GST	RX+GSH→HX+R-S-GSH

**Table 5:** Examples of Micro-/Nano-Particulate Delivery System Containing Polyphenols.

Delivery System	Polyphenol	Formulation Composition	Preparation Method	Remarks	Ref.
Microspheres 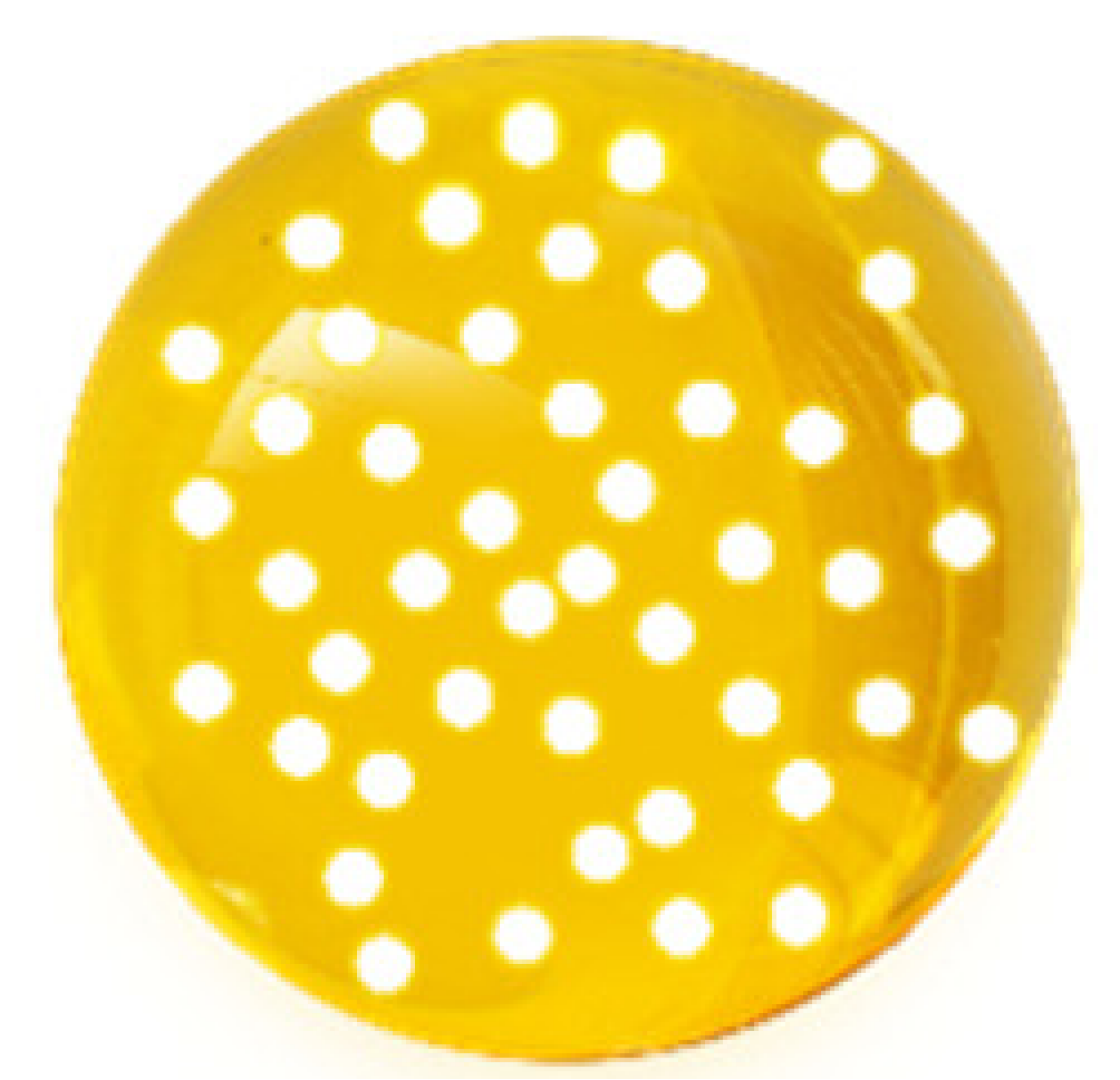 Microspheres are monolithic microcarriers with a diameter of 1–1000 μm	Ellagic acid	Liquid paraffin Span 80 Eudragit P-4135F	Solvent Evaporation	Ellagic acid was found to be protective against induced ulcerative colitis in rats and colon specific delivery was provided via microspheres.	[147]
	Pine Polyphenol	Chitosan	Emulsion Crosslinking	In vivo experiments on mice revealed improved protective effect against 60Co γ-radiation induced damages compared to free polyphenols due to improved stability and bioavailability via microspheres.	[148]
Coated Microparticles 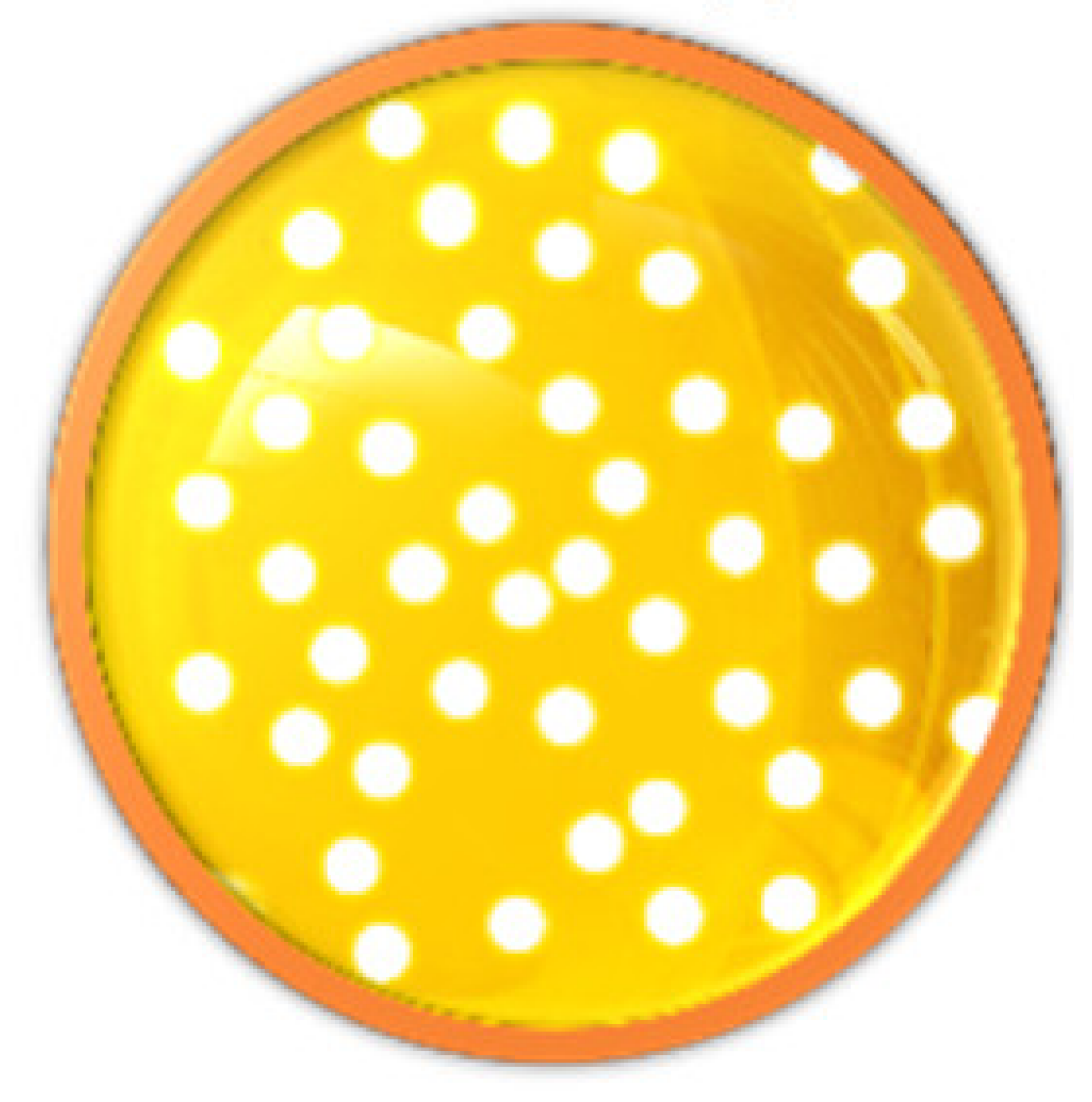 Microparticles coated with various materials (such as biodegradable, biocompatible polymers) to provide additional functionality	Resveratrol	Stearic acid Phosphatidylcholine Chitosan	Melt Emulsification	Directly nose to brain delivery of resveratrol was provided via chitosan coated lipid microparticles as demonstrated by increased drug concentration in cerebrospinal fluid in in vivo experiments.	[149]
	Curcumin	Zein Gantrez^®^ AN119 (PVMMA, Barcelona, Spain) Eudragit^®^ FS30D (Essen, Germany)	Spray Drying	Intestinal curcumin release and improved anti-inflammatory activity was shown by the inhibition of pro-inflammatory cytokines in macrophages. The microspheres were suggested for the treatment of inflammatory bowel diseases.	[150]
Microcapsules 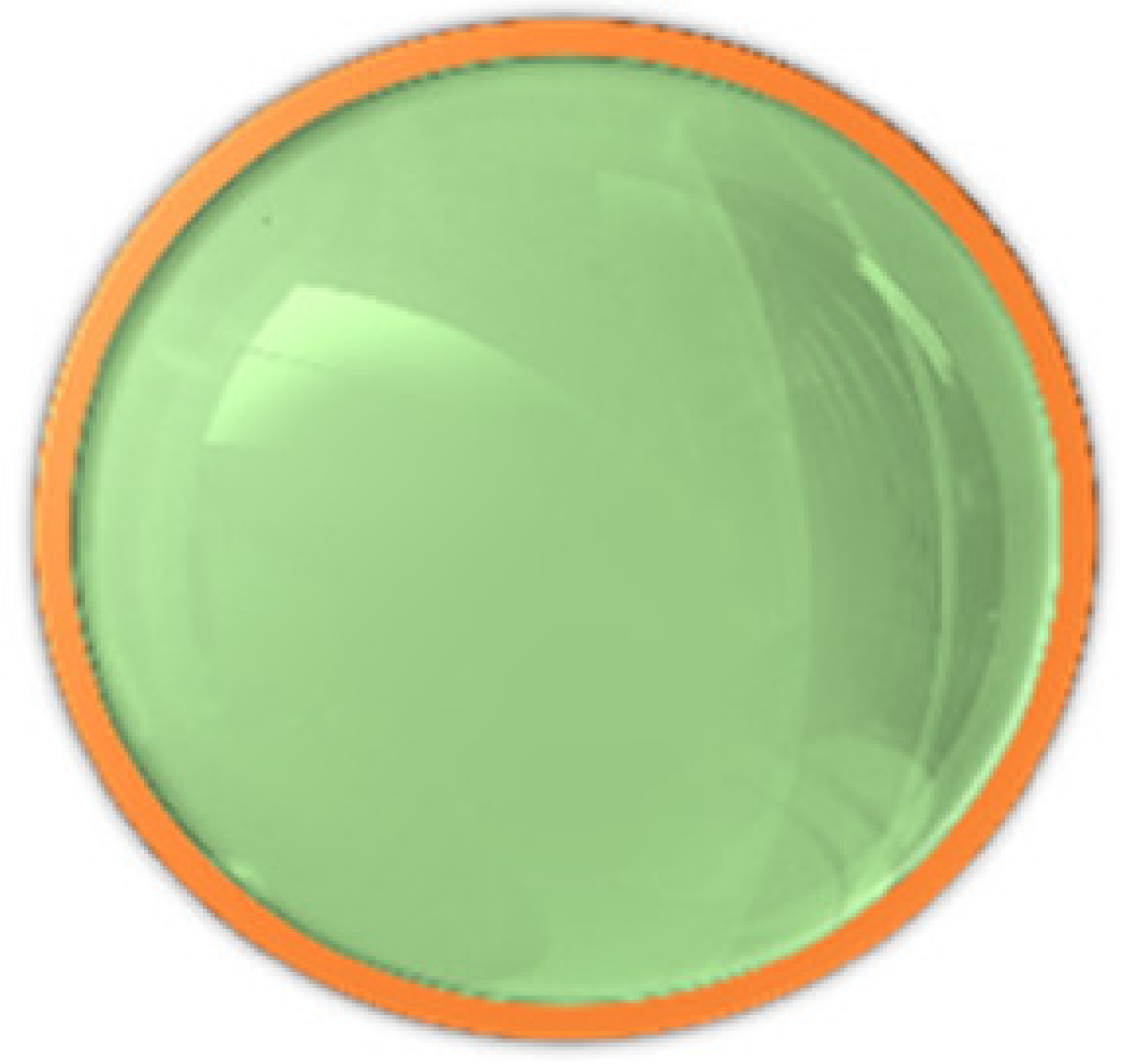 Microcapsules are delivery systems in which the reservoir is surrounded by a membrane shell that controls the release rate of the encapsulated cargo	Polyherbal Formulation (PHF) Extract	Gum arabic Gelatin Maltodextrin	Freeze Drying	Upon encapsulation antioxidant activity was successfully preserved. The microcapsules were found to have antidiabetic potential as α-amylase and α-glucosidase activities were inhibited, besides the in vivo studies on mice revealed improved bioavailability.	[151]
	Quercetin	Pectin Casein	Complex Coacervation-Spray Drying	Topical quercetin loaded microcapsules effectively blocked UVB induced alterations on mice. The anti-inflammatory effect of the prepared formulation was superior to free quercetin.	[152]
Polymeric nanoparticles 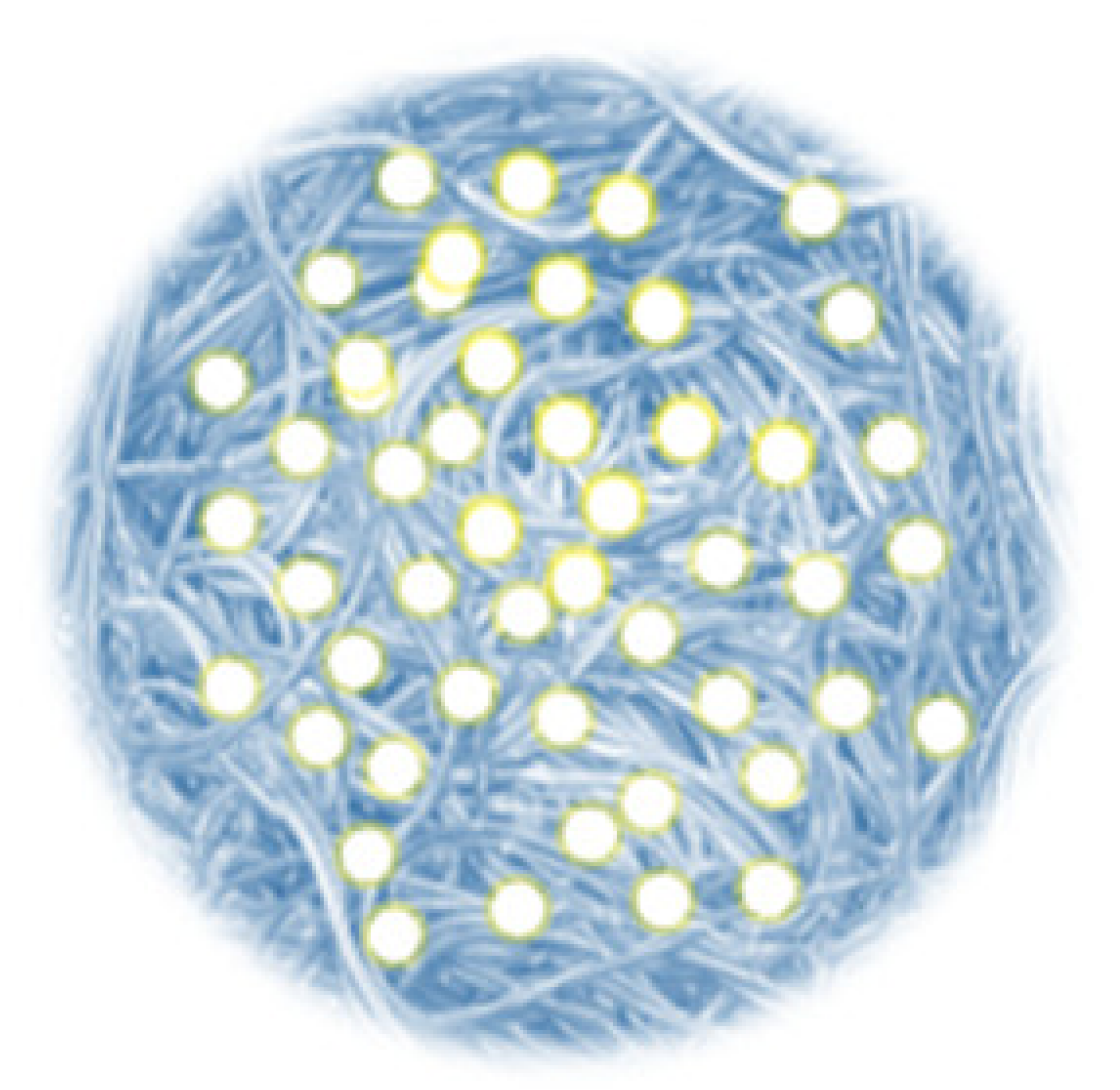 Nanoparticles are solid colloidal systems, usually prepared from biodegradable polymers, with diameters ranging from 10 to 1000 nm. They can be prepared in the form of nanocapsules or nanospheres	Silibinin	Palmitoyl chitosan Tripolyphosphate	Ionic Gelation Method	Enhanced cytotoxicity and controlled drug release was obtain via hydrophobic chitosan based silibinin nanoparticles	[153]
	Juglans regia *L. polyphenols*	Polysaccharides from Hohenbuehelia serotina	Solvent evaporation	Sustained polyphenol release and improved stability against gastrointestinal conditions was achieved by encapsulation of Juglans regia *L. polyphenols* in polysaccharide based nanoparticles	[154]
Solid lipid nanoparticles 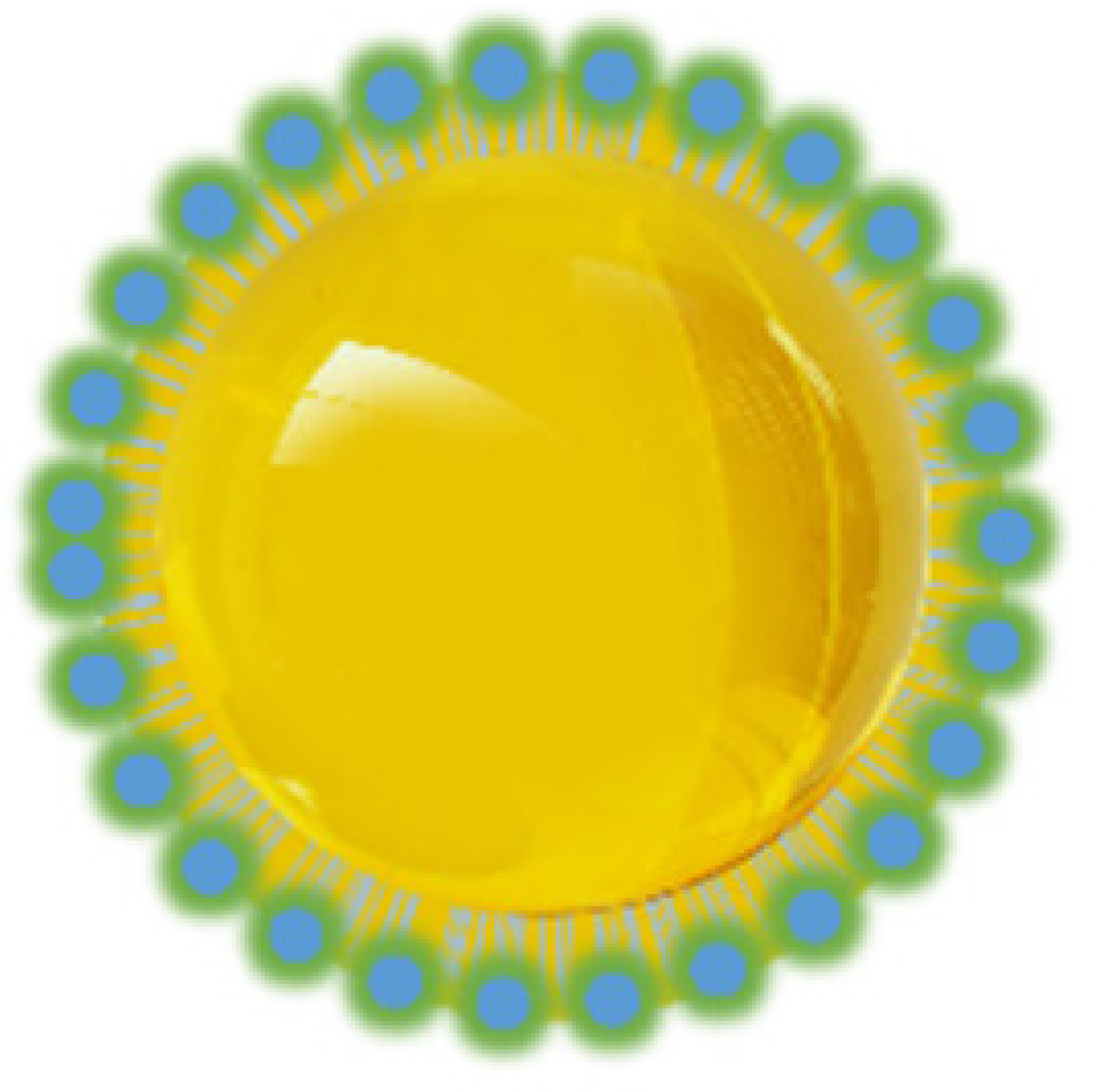 Nanocarriers that are composed of solid lipids at body temperature and stabilized by surfactants, have a size between 50–1000 nm and can be produced on a large scale	Pomegranate Extract	Stearic acid Lecithin Tween 80	Hot Homogenization and Ultra-Sonication	Cytotoxicity studies on MCF-7 cells showed that a 47-fold reduction in IC50 levels of pure pomegranate extract was achieved with solid lipid nanoparticles	[155]
	Diosmin	Compritol ATO 888 Soy lecithin Tween 80	Hot Homogenization and Freeze Drying	Diosmin loaded solid lipid nanoparticles were superior to free diosmin in terms of antioxidant activity. The in vivo hepatic tumor incidence was also lower.	[156]
Liposomes 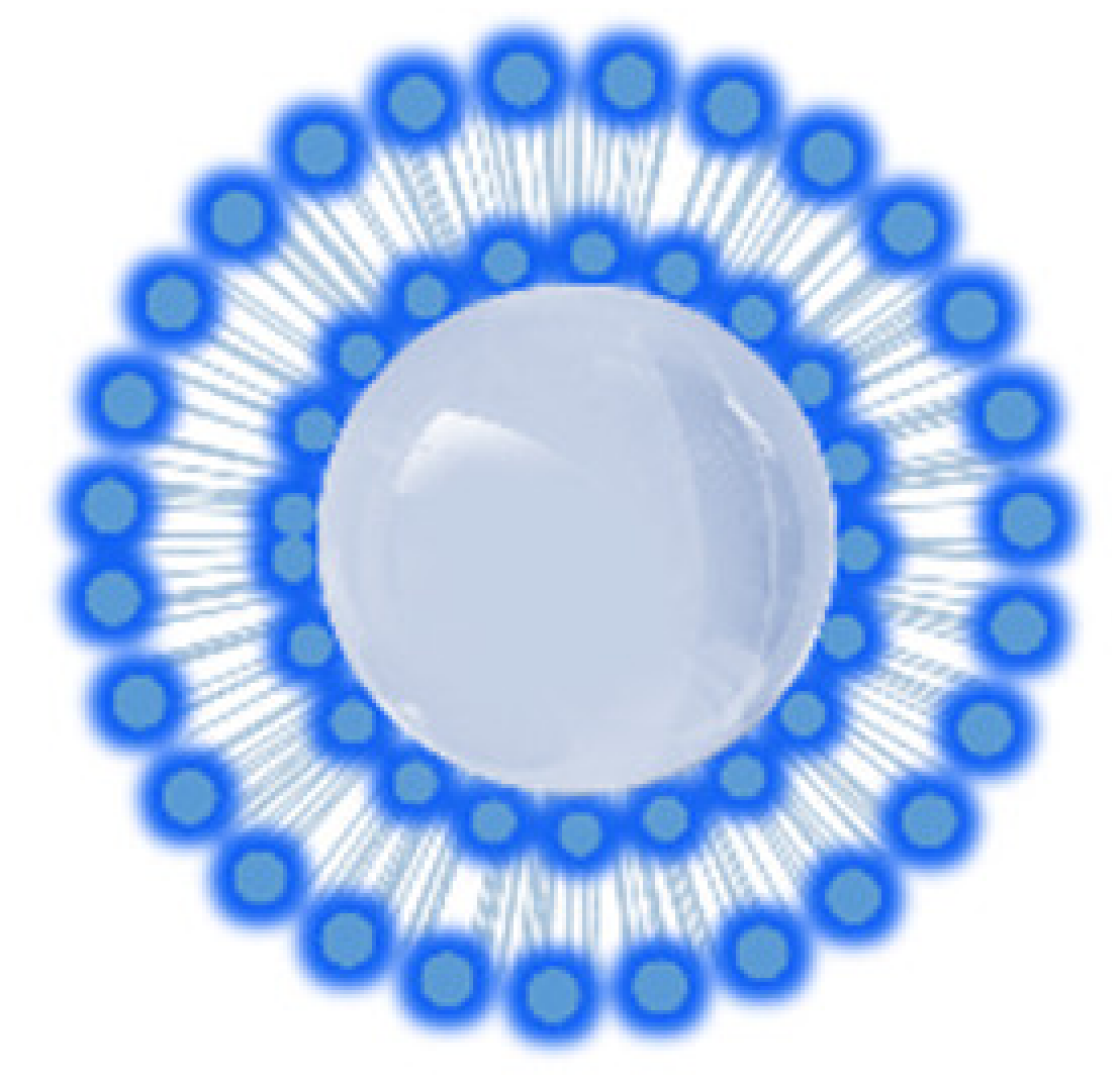 Phospholipid based vesicular structures which can carry hydrophilic, amphiphilic, or lipophilic compounds	Resveratrol	EggPC DSPE-PEG2000, DOPE CHEMS Tf-PEG3400-PE	Thin Film Hydration Method	Transferin targeted liposomes significantly improved the tumor growth inhibition potential of resveratrol and survival in glioblastoma-bearing mice.	[157]
	Quercetin	Phosphatidylcholine Stearylamine Eudragit	Sonication	Eudragit coated liposomes improved the physical stability of quercetin in gastrointestinal conditions and showed prolonged antioxidant effect on intestinal HT-29 cells	[158]
Polymeric micelles 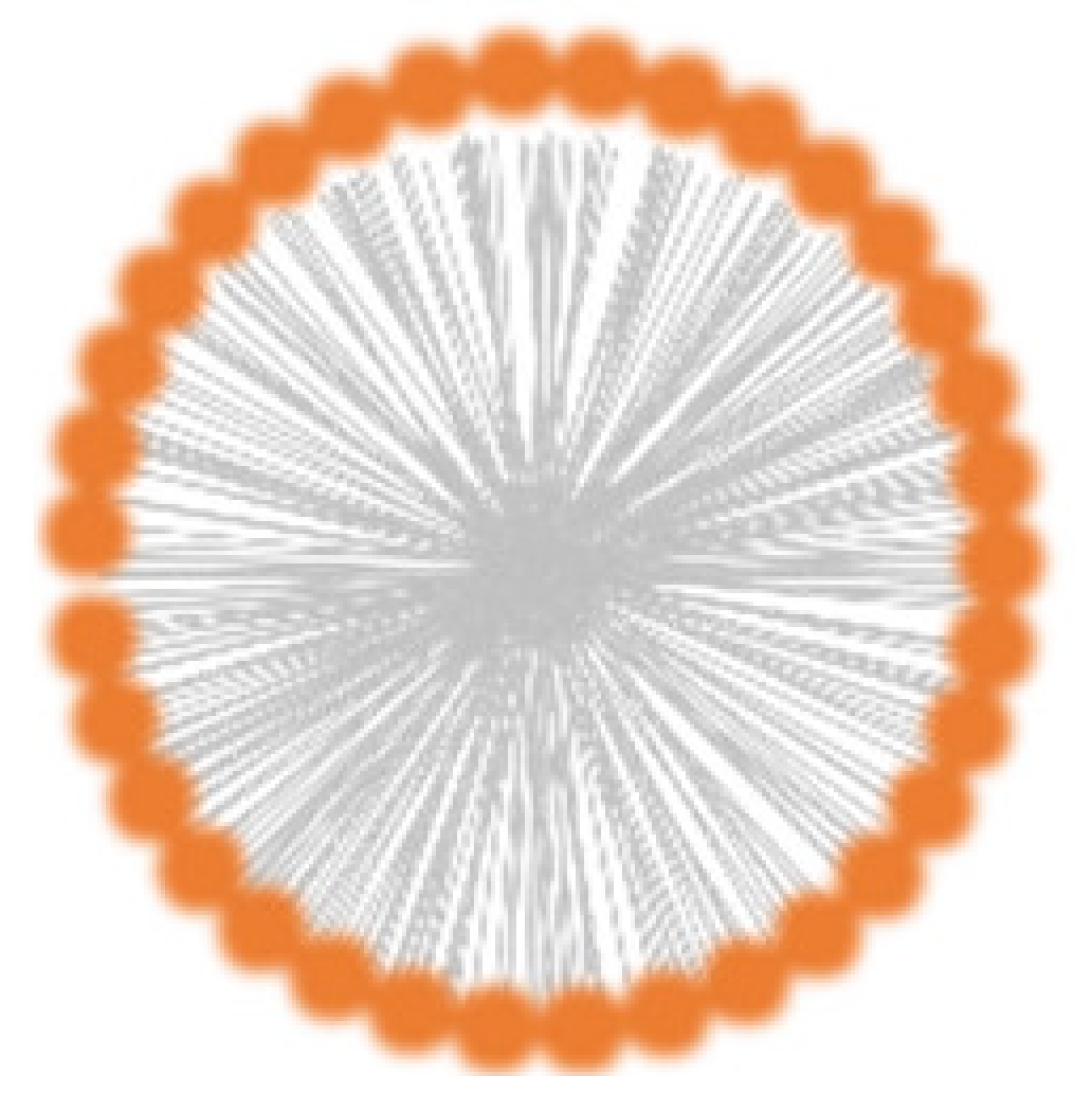 Polymeric micelles are spherical core-corona aggregates of amphiphilic copolymers and their size is in the range of 10–100 nm	Resveratrol and Quercetin	Pluronic F127	Thin Film Hydration Method	Resveratrol and quercetin loaded polymeric micelles were effective on reducing doxorubicin hydrochloride induced cardiotoxicity in mice.	[159]
	Syringic acid	TPGS Pluronic F127 Pluronic F68	Thin Film Hydration Method	In vivo pharmacokinetic tests on rats revealed improved oral bioavailability of syringic acid after liposomal encapsulation. Enhanced cellular internalization was shown on HepG2 cells and liver targeting properties of mixed micelles was shown. Overall formulation was found to be promising for the utilization of syringic acid in functional foods.	[160]

**Table 6:** Inclusion constants *K_AO_* for gallates and values of the concentration of gallates required to reduce the DPPH^•^ radical concentration by 50% (EC_50_ value) [178].

AO	K_AO_ (M^−1^)		10^5^ EC_50_ (M)	
	β-CD	HP-β-CD	0 M β-CD	11 mM β-CD
Gallic acid	-	551	6.5 ± 0.1	5.8 ± 0.1
Methyl gallate	-	595		
Ethyl gallate	-	380		
Propyl gallate (PG)	125 ± 11 ^a^ 105 ± 2 ^b^	343 ± 9 ^a^ 328 ± 7 ^b^	5.7 ± 0.1	3.9 ± 0.1
Butyl gallate (BG)	198 ± 13 ^a^ 200 ± 15 ^b^	706 ± 38 ^a^	5.2 ± 0.3	4.1 ± 0.2
Octyl gallate (OG)		4810 ± 98 ^b^	5.7 ± 0.1	4.1 ± 0.1

Values from reference [32] obtained by ^a^ UV shift method, ^b^ phase-solubility method (buffered aqueous solution with citric acid-sodium citrate 0.04 M, pH 3.65).

## Data Availability

Not applicable.
